# Supplementary material for: Universal healthcare coverage and health service delivery before and during the COVID-19 pandemic: A difference-in-difference study of childhood immunization coverage from 195 countries
Source: PLoS Med. 2022 Aug 16;19(8):e1004060. doi: 10.1371/journal.pmed.1004060 (PMC9380914; doi:10.1371/journal.pmed.1004060)
Supplement: S1 Text — (DOCX) [file pmed.1004060.s001.docx]

**S1 Text. References to the dataset used for the analysis**

Full dataset used for this analysis can be found here: <https://github.com/sk9076/UHC_DID>

References to the original data

1. Global Health Security Index. Global Health Security (GHS) Index 2019. [Available from: https://www ghsindex org/wp-content/uploads/2019/10/2019-Global-Health-Security-Index pdf.2.

2. Institute for Health Metrics and Evaluation. Global Burden of Disease Study 2019 (GBD 2019) UHC Effective Coverage Index 1990-2019. In:2020. [Available from: https://ghdx.healthdata.org/record/ihme-data/gbd-2019-uhc-effective-coverage-index-1990-2019.

3. UNICEF. Immunization. In:2021. [Available from*:* https://data.unicef.org/topic/child-health/immunization/.

4. World Bank. The World by Income and Region. [Available from: https://datatopics.worldbank.org/world-development-indicators/the-world-by-income-and-region.html. Published 2021.
