# Supplementary material for: Universal healthcare coverage and health service delivery before and during the COVID-19 pandemic: A difference-in-difference study of childhood immunization coverage from 195 countries
Source: PLoS Med. 2022 Aug 16;19(8):e1004060. doi: 10.1371/journal.pmed.1004060 (PMC9380914; doi:10.1371/journal.pmed.1004060)
Supplement: S1 File — Paragraph A. Data source, compiled dataset, link to the repository. Table A. Complete list of 195 countries included in the dataset with their UHC SCI 2019 in alphabetical. Fig A. Change in overall vaccine coverage (A) and vaccine specific coverage (B) globally by UHC Service Coverage Index 2019 (UHC SCI <50 vs. UHC SCI ≥50) between 1997 and 2020. Table B. Results of ordinary-square linear regression analysis to assess the parallel pre-trend assumption before the COVID-19 pandemic. Fig B. Histogram of the distribution of UHC SCI 2019 with the cutoff value of 80 for the treatment vs. control group marked with dotted line. Table C. Summary characteristics of the countries included in the analysis (N = 195) based on the cutoff value of 50 for the UHC Service Coverage Index 2019. (DOCX) [file pmed.1004060.s002.docx]

**Universal Healthcare Coverage and Health Service Delivery in Times of Public Health Crises: A Difference-in-Difference Study of Childhood Immunization Coverage from 195 Countries Before and During the COVID-19 Pandemic**

**Supporting Information 1**

**Table A. Complete list of 195 countries included in the dataset with their UHC SCI 2019 in alphabetical order**

| Country name | UHC SCI 2019 |
| --- | --- |
| Afghanistan | 39.2945 |
| Albania | 69.6255 |
| Algeria | 64.8553 |
| Andorra | 91.7482 |
| Angola | 39.1577 |
| Antigua and Barbuda | 59.6303 |
| Argentina | 61.1584 |
| Armenia | 62.4351 |
| Australia | 89.4234 |
| Austria | 86.3701 |
| Azerbaijan | 48.1814 |
| Bahamas | 60.5651 |
| Bahrain | 70.5773 |
| Bangladesh | 53.8827 |
| Barbados | 61.2069 |
| Belarus | 70.4622 |
| Belgium | 87.3014 |
| Belize | 54.2797 |
| Benin | 44.6243 |
| Bhutan | 51.3014 |
| Bolivia, Plurinational State of | 52.3988 |
| Bosnia and Herzegovina | 64.1839 |
| Botswana | 57.5176 |
| Brazil | 64.8276 |
| Brunei Darussalam | 65.532 |
| Bulgaria | 62.5567 |
| Burkina Faso | 41.7968 |
| Burundi | 49.94 |
| Cabo Verde | 62.188 |
| Cambodia | 57.0794 |
| Cameroon | 42.29 |
| Canada | 90.3017 |
| Central African Republic | 22.2999 |
| Chad | 31.3724 |
| Chile | 74.3496 |
| China | 69.7123 |
| Colombia | 74.3969 |
| Comoros | 48.1389 |
| Congo | 43.9038 |
| Cook Islands | 62.2277 |
| Costa Rica | 79.015 |
| Cote d’Ivoire | 43.0401 |
| Croatia | 78.9352 |
| Cuba | 72.5922 |
| Cyprus | 79.6022 |
| Czechia | 81.944 |
| Democratic People’s Republic of Korea | 52.8378 |
| Democratic Republic of the Congo | 45.1687 |
| Denmark | 84.1397 |
| Djibouti | 45.2867 |
| Dominica | 51.8089 |
| Dominican Republic | 52.4998 |
| Ecuador | 64.4544 |
| Egypt | 54.7965 |
| El Salvador | 61.6782 |
| Equatorial Guinea | 49.9937 |
| Eritrea | 42.275 |
| Estonia | 82.0393 |
| Eswatini | 53.3968 |
| Ethiopia | 46.5216 |
| Fiji | 45.1761 |
| Finland | 91.3486 |
| France | 90.7662 |
| Gabon | 53.0498 |
| Gambia | 48.065 |
| Georgia | 55.9534 |
| Germany | 86.2487 |
| Ghana | 49.1393 |
| Greece | 80.1403 |
| Grenada | 50.4838 |
| Guatemala | 52.0988 |
| Guinea | 32.3346 |
| Guinea Bissau | 35.7073 |
| Guyana | 40.6219 |
| Haiti | 35.8119 |
| Honduras | 54.2843 |
| Hungary | 72.0281 |
| Iceland | 95.3069 |
| India | 46.8261 |
| Indonesia | 48.7277 |
| Iran, Islamic Republic of | 69.5146 |
| Iraq | 57.7248 |
| Ireland | 90.3459 |
| Israel | 81.3846 |
| Italy | 88.895 |
| Jamaica | 56.8395 |
| Japan | 96.3409 |
| Jordan | 69.9667 |
| Kazakhstan | 59.2368 |
| Kenya | 51.6469 |
| Kiribati | 35.7356 |
| Kuwait | 81.8333 |
| Kyrgyzstan | 52.9515 |
| Lao People’s Democratic Republic | 43.8545 |
| Latvia | 69.7884 |
| Lebanon | 74.5332 |
| Lesotho | 38.7373 |
| Liberia | 47.5997 |
| Libya | 66.3277 |
| Lithuania | 70.3524 |
| Luxembourg | 91.4552 |
| Madagascar | 39.6908 |
| Malawi | 55.5208 |
| Malaysia | 66.5736 |
| Maldives | 66.8566 |
| Mali | 40.6607 |
| Malta | 82.8809 |
| Marshall Islands | 44.0043 |
| Mauritania | 53.2779 |
| Mauritius | 55.8094 |
| Mexico | 61.4368 |
| Micronesia, Federated States of | 34.4743 |
| Monaco | 91.3533 |
| Mongolia | 47.907 |
| Montenegro | 65.9585 |
| Morocco | 58.0323 |
| Mozambique | 44.044 |
| Myanmar | 46.9541 |
| Namibia | 62.169 |
| Nauru | 42.0017 |
| Nepal | 47.2804 |
| Netherlands | 89.5884 |
| New Zealand | 82.9777 |
| Nicaragua | 57.1586 |
| Niger | 35.0256 |
| Nigeria | 38.339 |
| Niue | 49.0104 |
| North Macedonia | 60.7461 |
| Norway | 94.2406 |
| Oman | 71.2208 |
| Pakistan | 39.1679 |
| Palau | 45.0665 |
| Palestine | 61.2338 |
| Panama | 71.1553 |
| Papua New Guinea | 37.7673 |
| Paraguay | 63.3513 |
| Peru | 75.7594 |
| Philippines | 54.7117 |
| Poland | 72.6564 |
| Portugal | 83.533 |
| Qatar | 80.4034 |
| Republic of Korea | 89.1617 |
| Republic of Moldova | 62.1936 |
| Romania | 69.5851 |
| Russian Federation | 68.9736 |
| Rwanda | 59.3591 |
| Saint Kitts and Nevis | 52.8848 |
| Saint Lucia | 59.1403 |
| Saint Vincent and the Grenadines | 49.4939 |
| Samoa | 49.7959 |
| San Marino | 92.7161 |
| Sao Tome and Principe | 54.7554 |
| Saudi Arabia | 64.1955 |
| Senegal | 49.6099 |
| Serbia | 63.3494 |
| Seychelles | 61.5172 |
| Sierra Leone | 42.1196 |
| Singapore | 92.4397 |
| Slovakia | 77.9829 |
| Slovenia | 89.834 |
| Solomon Islands | 39.3331 |
| Somalia | 23.9397 |
| South Africa | 59.7274 |
| South Sudan | 41.6941 |
| Spain | 90.0056 |
| Sri Lanka | 65.5627 |
| Sudan | 51.8347 |
| Suriname | 50.1342 |
| Sweden | 90.3608 |
| Switzerland | 93.4984 |
| Syrian Arab Republic | 57.5646 |
| Tajikistan | 47.8775 |
| Thailand | 71.6003 |
| Timor Leste | 45.9543 |
| Togo | 42.8086 |
| Tonga | 52.4167 |
| Trinidad and Tobago | 55.5174 |
| Tunisia | 68.1057 |
| Turkey | 69.2104 |
| Turkmenistan | 44.0134 |
| Tuvalu | 39.5697 |
| Uganda | 52.7479 |
| Ukraine | 56.7521 |
| United Arab Emirates | 63.3574 |
| United Kingdom | 87.9 |
| United Republic of Tanzania | 55.2493 |
| United States | 82.1377 |
| Uruguay | 68.5299 |
| Uzbekistan | 42.1848 |
| Vanuatu | 34.0835 |
| Venezuela, Bolivarian Republic of | 60.9675 |
| Viet Nam | 59.7068 |
| Yemen | 49.0458 |
| Zambia | 52.6937 |
| Zimbabwe | 54.4611 |


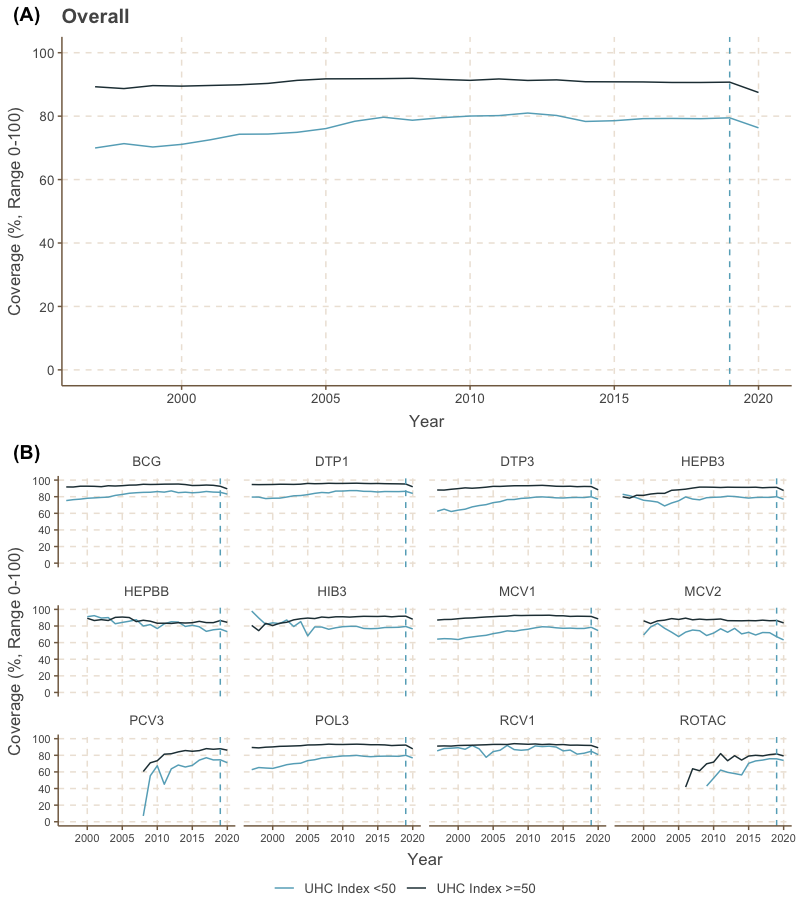


**Figure A.** Change in overall vaccine coverage (A) and vaccine specific coverage (B) globally by UHC Service Coverage Index 2019 (UHC SCI <50 vs. UHC SCI ≥50) between 1997 and 2020

**Table B. Results of ordinary-square linear regression analysis to assess the parallel pre-trend assumption before the COVID-19 pandemic**

| **Characteristic** | **Beta** | **95% CI**^a^ | ***p-value*** |
| --- | --- | --- | --- |
| Intercept | -207 | -262, -152 | <0.001 |
| Year | 0.15 | 0.12, 0.17 | <0.001 |
| Treatment (UHC SCI 2019 ≥80) | 71.5 | -56.9, 200 | 0.28 |
| Year * Treatment | -0.03 | -0.10, 0.03 | 0.32 |

| a: CI = Confidence Interval |
| --- |


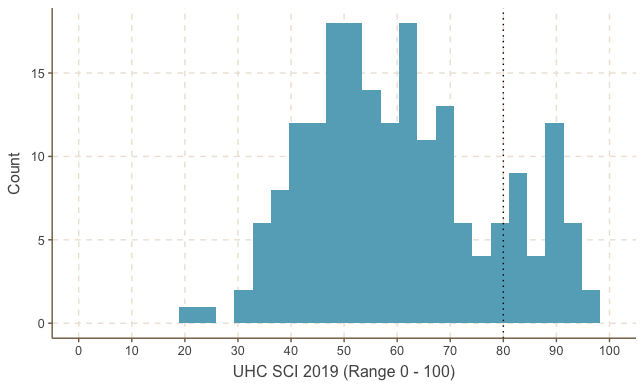


**Figure B. Histogram of the distribution of UHC SCI 2019 with the cutoff value of 80 for the treatment vs. control group marked with dotted line**

**Table C. Summary characteristics of the countries included in the analysis (N=195) based on the cut-off value of 50 for the UHC Service Coverage Index 2019**

|  | **UHC Index <50 (N=60)** | **UHC Index >=50 (N=135)** | **Overall (N=195)** |
| --- | --- | --- | --- |
| **UHC SCI**^a^ **2019** |  |  |  |
| Mean (SD^b^) | 42.4 (6.07) | 68.7 (13.4) | 60.6 (16.8) |
| **World Bank Income group** |  |  |  |
| High | 3 (5.0%) | 58 (43.0%) | 61 (31.3%) |
| Upper-Middle | 10 (16.7%) | 44 (32.6%) | 54 (27.7%) |
| Lower-Middle | 23 (38.3%) | 26 (19.3%) | 49 (25.1%) |
| Low | 24 (40.0%) | 6 (4.4%) | 30 (15.4%) |
| **WHO Region** |  |  |  |
| Africa | 29 (48.3%) | 18 (13.3%) | 47 (24.1%) |
| Americas | 3 (5.0%) | 32 (23.7%) | 35 (17.9%) |
| Eastern Mediterranean | 5 (8.3%) | 17 (12.6%) | 22 (11.3%) |
| Europe | 4 (6.7%) | 49 (36.3%) | 53 (27.2%) |
| South East Asia | 5 (8.3%) | 6 (4.4%) | 11 (5.6%) |
| Western Pacific | 14 (23.3%) | 13 (9.6%) | 27 (13.8%) |
| **Global Health Security Index 2019** |  |  |  |
| Mean (SD^b^) | 30.2 (8.27) | 44.7 (14.6) | 40.2 (14.6) |

a**:** UHC SCI: Universal Health Coverage Service Coverage Index

b: Standard deviation
