# Supplementary material for: Universal healthcare coverage and health service delivery before and during the COVID-19 pandemic: A difference-in-difference study of childhood immunization coverage from 195 countries
Source: PLoS Med. 2022 Aug 16;19(8):e1004060. doi: 10.1371/journal.pmed.1004060 (PMC9380914; doi:10.1371/journal.pmed.1004060)
Supplement: S2 File — Table A. Regression analysis results of countries with high UHC index values (UHC SCI ≥80) vs. all other countries (UHC SCI <80) in childhood immunization coverage in pre-pandemic period (2010–2019) compared with pandemic period (2020)—Base model with no DiD interaction term (Unadjusted). Table B. Difference-in-difference regression analysis of bacille Calmette–Guérin (BCG) coverage after COVID-19 pandemic by UHC SCI 2019 (≥80 vs. the rest)—Unadjusted. Table C. Difference-in-difference regression analysis of bacille Calmette–Guérin (BCG) coverage after COVID-19 pandemic by UHC SCI 2019 (≥80 vs. the rest)—Adjusted for calendar year, pandemic preparedness, country income group, and geographic region. Table D. Difference-in-difference regression analysis of the first dose of diphtheria and tetanus toxoid and pertussis containing vaccine (DTP1) coverage after COVID-19 pandemic by UHC SCI 2019 (≥80 vs. the rest)—Unadjusted. Table E. Difference-in-difference regression analysis of the first dose of diphtheria and tetanus toxoid and pertussis containing vaccine (DTP1) coverage after COVID-19 pandemic by UHC SCI 2019 (≥80 vs. the rest)—Adjusted for calendar year, pandemic preparedness, country income group, and geographic region. Table F. Difference-in-difference regression analysis of the third dose of diphtheria and tetanus toxoid and pertussis containing vaccine (DTP3) coverage after COVID-19 pandemic by UHC SCI 2019 (≥80 vs. the rest)—Unadjusted. Table G. Difference-in-difference regression analysis of the third dose of diphtheria and tetanus toxoid and pertussis containing vaccine (DTP3) coverage after COVID-19 pandemic by UHC SCI 2019 (≥80 vs. the rest)—Adjusted for calendar year, pandemic preparedness, country income group, and geographic region. Table H. Difference-in-difference regression analysis of the third dose of hepatitis B containing vaccine (HEPB3) coverage after COVID-19 pandemic by UHC SCI 2019 (≥80 vs. the rest)—Unadjusted. Table I. Difference-in-differe [file pmed.1004060.s003.docx]

**Universal Healthcare Coverage and Health Service Delivery in Times of Public Health Crises: A Difference-in-Difference Study of Childhood Immunization Coverage from 195 Countries Before and During the COVID-19 Pandemic**

**Supporting Information 2**

**Table A. Regression analysis results of countries with high UHC index values (UHC SCI ≥80) vs. all other countries (UHC SCI <80) in childhood immunization coverage in**

**pre-pandemic period (2010-2019) compared with pandemic period (2020) – Base model with no DiD interaction term (Unadjusted)**

|  | Base model (unadjusted) | | | Difference-in-difference model (unadjusted) | | |
| --- | --- | --- | --- | --- | --- | --- |
| Variable | Coefficient | 95% CI^a^ | p-value | Coefficient | 95% CI^a^ | p-value |
| Intercept | 86.53 | (86.30, 86.77) | <0.001 | 86.58 | (86.34, 86.82) | <0.001 |
| Pre/Post | -3.78 | (-4.54, -3.02) | <0.001 | -4.3 | (-5.12, -3.48) | <0.001 |
| UHC SCI^b^ 2019 ≥80 | 6.3 | (5.75, 6.85) | <0.001 | 6.05 | (5.47, 6.62) | <0.001 |
| Pre/Post * UHC SCI^b^ ≥80 |  |  |  | 3.63 | (1.46, 5.80) | 0.001 |

a: Confidence Interval

b: UHC Service Coverage Index

**Table B. Difference-in-difference regression analysis of Bacille Calmette-Guérin (BCG) coverage after COVID-19 pandemic by UHC SCI 2019 (≥80 vs. the rest) – Unadjusted**

|  | Base model | | | Difference-in-difference model | | |
| --- | --- | --- | --- | --- | --- | --- |
| Variable | Coefficient | 95% CI^a^ | p-value | Coefficient | 95% CI^a^ | p-value |
| Intercept | 91.44 | (90.80, 92.07) | <0.001 | 85.6 | (84.64, 86.57) | <0.001 |
| Pre/Post | -4.34 | (-6.56, -2.12) | <0.001 | -2.47 | (-5.80, 0.86) | 0.146 |
| UHC SCI^b^ 2019 ≥80 | -7.67 | (-10.32, -5.01) | <0.001 | 8.65 | (7.43, 9.86) | <0.001 |
| Pre/Post * UHC SCI^b^ ≥80 |  |  |  | -2.33 | (-6.65, 1.99) | 0.291 |

a: Confidence Interval

b: UHC Service Coverage Index

**Table C. Difference-in-difference regression analysis of Bacille Calmette-Guérin (BCG) coverage after COVID-19 pandemic by UHC SCI 2019 (≥80 vs. the rest) – Adjusted for calendar year, pandemic preparedness, country income group, and geographic region**

|  | Base model | | | Difference-in-difference model | | |
| --- | --- | --- | --- | --- | --- | --- |
| Variable | Coefficient | 95% CI^a^ | p-value | Coefficient | 95% CI^a^ | p-value |
| Intercept | 511.42 | (109.35, 913.49) | 0.013 | 510.91 | (108.73, 913.10) | 0.013 |
| Year | -0.21 | (-0.41, -0.01) | 0.037 | -0.21 | (-0.41, -0.01) | 0.037 |
| GHSI^b^ 2019 | 0 | (-0.06, 0.06) | 0.977 | 0 | (-0.06, 0.06) | 0.977 |
| World Bank Income Group (Reference category: Low) | | | | | | |
| Lower-middle | 7.87 | (6.14, 9.60) | <0.001 | 7.87 | (6.14, 9.60) | <0.001 |
| Upper-middle | 12.26 | (10.33, 14.20) | <0.001 | 12.26 | (10.33, 14.20) | <0.001 |
| High | 15.14 | (12.78, 17.49) | <0.001 | 15.14 | (12.78, 17.50) | <0.001 |
| WHO Region (Reference category: Americas) | | | | | | |
| Europe | -0.78 | (-2.68, 1.12) | 0.423 | -0.77 | (-2.68, 1.13) | 0.425 |
| Western Pacific | 0.97 | (-1.08, 3.02) | 0.354 | 0.97 | (-1.08, 3.02) | 0.355 |
| Eastern Mediterranean | -1.29 | (-3.48, 0.89) | 0.245 | -1.29 | (-3.48, 0.89) | 0.246 |
| Southeast Asia | 4.23 | (1.66, 6.80) | 0.001 | 4.23 | (1.66, 6.80) | 0.001 |
| Africa | 0 | (-1.95, 1.95) | 0.999 | 0 | (-1.95, 1.95) | 1 |
| Difference-in-difference variables | | | | | | |
| Pre/Post | -2.81 | (-5.14, -0.48) | 0.018 | -2.74 | (-5.11, -0.38) | 0.023 |
| UHC SCI^c^ 2019 ≥80 | -13.62 | (-16.65, -10.59) | <0.001 | -13.52 | (-16.60, -10.45) | <0.001 |
| Pre/Post * UHC SCI^c^ ≥80 |  |  |  | -2.19 | (-13.94, 9.57) | 0.716 |

a: Confidence Interval

b: Global Health Security Index

c: UHC Service Coverage Index

**Table D. Difference-in-difference regression analysis of the first dose of diphteria and tetanus toxoid and pertussis containing vaccine (DTP1) coverage after COVID-19 pandemic by UHC SCI 2019 (**≥**80 vs. the rest) - Unadjusted**

|  | Base model | | | Difference-in-difference model | | |
| --- | --- | --- | --- | --- | --- | --- |
| Variable | Coefficient | 95% CI^a^ | p-value | Coefficient | 95% CI^a^ | p-value |
| Intercept | 91.93 | (91.46, 92.40) | <0.001 | 86.5 | (85.78, 87.22) | <0.001 |
| Pre/Post | -3.58 | (-5.15, -2.02) | <0.001 | -2.54 | (-5.01, -0.06) | 0.045 |
| UHC SCI^b^ 2019 ≥80 | 5.67 | (4.58, 6.75) | <0.001 | 9.31 | (8.44, 10.17) | <0.001 |
| Pre/Post * UHC SCI^b^ ≥80 |  |  |  | -1.34 | (-4.39, 1.71) | 0.39 |

a: Confidence Interval

b: UHC Service Coverage Index

**Table E. Difference-in-difference regression analysis of the first dose of diphteria and tetanus toxoid and pertussis containing vaccine (DTP1) coverage after COVID-19 pandemic by UHC SCI 2019 (**≥**80 vs. the rest) – Adjusted for calendar year, pandemic preparedness, country income group, and geographic region**

|  | Base model | | | Difference-in-difference model | | |
| --- | --- | --- | --- | --- | --- | --- |
| Variable | Coefficient | 95% CI^a^ | p-value | Coefficient | 95% CI^a^ | p-value |
| Intercept | 283.85 | (14.42, 553.28) | 0.039 | 283.9 | (14.51, 553.30) | 0.039 |
| Year | -0.1 | (-0.23, 0.03) | 0.144 | -0.1 | (-0.23, 0.03) | 0.144 |
| GHSI 2019^b^ | 0.06 | (0.02, 0.09) | 0.001 | 0.06 | (0.02, 0.09) | 0.001 |
| World Bank Income Group (Reference category: Low) | | | | | | |
| Lower-middle | 6.03 | (4.75, 7.32) | <0.001 | 6.03 | (4.75, 7.32) | <0.001 |
| Upper-middle | 9.23 | (7.81, 10.65) | <0.001 | 9.23 | (7.81, 10.64) | <0.001 |
| High | 12.76 | (11.15, 14.37) | <0.001 | 12.75 | (11.14, 14.36) | <0.001 |
| WHO Region (Reference category: Americas) | | | | | | |
| Europe | 1.15 | (-0.08, 2.38) | 0.067 | 1.15 | (-0.08, 2.37) | 0.067 |
| Western Pacific | 0.35 | (-1.02, 1.73) | 0.616 | 0.36 | (-1.02, 1.73) | 0.613 |
| Eastern Mediterranean | -2.02 | (-3.49, -0.55) | 0.007 | -2.01 | (-3.49, -0.54) | 0.007 |
| Southeast Asia | 3.16 | (1.30, 5.02) | 0.001 | 3.16 | (1.30, 5.02) | 0.001 |
| Africa | -1.29 | (-2.66, 0.09) | 0.067 | -1.29 | (-2.66, 0.09) | 0.066 |
| Difference-in-difference variables | | | | | | |
| Pre/Post | -2.64 | (-4.22, -1.07) | 0.001 | -3.02 | (-4.70, -1.34) | <0.001 |
| UHC SCI^c^ 2019 ≥80 | -2.18 | (-3.70, -0.66) | 0.005 | -2.33 | (-3.87, -0.80) | 0.003 |
| Pre/Post * UHC SCI^c^ ≥80 |  |  |  | 2.46 | (-1.41, 6.32) | 0.213 |

a: Confidence Interval

b: Global Health Security Index

c: UHC Service Coverage Index

**Table F. Difference-in-difference regression analysis of the third dose of diphteria and tetanus toxoid and pertussis containing vaccine (DTP3) coverage after COVID-19 pandemic by UHC SCI 2019 (≥80 vs. the rest) - Unadjusted**

|  | Base model | | | Difference-in-difference model | | |
| --- | --- | --- | --- | --- | --- | --- |
| Variable | Coefficient | 95% CI^a^ | p-value | Coefficient | 95% CI^a^ | p-value |
| Intercept | 87.05 | (86.42, 87.68) | <0.001 | 79.1 | (78.16, 80.05) | <0.001 |
| Pre/Post | -3.89 | (-5.98, -1.80) | <0.001 | -2.09 | (-5.35, 1.18) | 0.21 |
| UHC SCI^b^ 2019 ≥80 | 8.51 | (7.06, 9.96) | <0.001 | 13.68 | (12.54, 14.81) | <0.001 |
| Pre/Post * UHC SCI^b^ ≥80 |  |  |  | -2.38 | (-6.40, 1.64) | 0.246 |

a: Confidence Interval

b: UHC Service Coverage Index

**Table G. Difference-in-difference regression analysis of the third dose of diphteria and tetanus toxoid and pertussis containing vaccine (DTP3) coverage after COVID-19 pandemic by UHC SCI 2019 (≥80 vs. the rest) – Adjusted for calendar year, pandemic preparedness, country income group, and geographic region**

|  | Base model | | | Difference-in-difference model | | |
| --- | --- | --- | --- | --- | --- | --- |
| Variable | Coefficient | 95% CI^a^ | p-value | Coefficient | 95% CI^a^ | p-value |
| Intercept | 277.68 | (-77.89, 633.24) | 0.126 | 277.73 | (-77.85, 633.31) | 0.126 |
| Year | -0.1 | (-0.28, 0.08) | 0.26 | -0.1 | (-0.28, 0.08) | 0.26 |
| GHSI^b^ 2019 | 0.1 | (0.05, 0.15) | <0.001 | 0.1 | (0.05, 0.15) | <0.001 |
| World Bank Income Group (Reference category: Low) | | | | | | |
| Lower-middle | 8.4 | (6.71, 10.09) | <0.001 | 8.4 | (6.71, 10.09) | <0.001 |
| Upper-middle | 12.33 | (10.46, 14.20) | <0.001 | 12.33 | (10.45, 14.20) | <0.001 |
| High | 17.52 | (15.39, 19.64) | <0.001 | 17.51 | (15.38, 19.64) | <0.001 |
| WHO Region (Reference category: Americas) | | | | | | |
| Europe | 2.11 | (0.49, 3.73) | 0.011 | 2.11 | (0.49, 3.73) | 0.011 |
| Western Pacific | 0.17 | (-1.64, 1.99) | 0.851 | 0.18 | (-1.64, 1.99) | 0.849 |
| Eastern Mediterranean | -1.35 | (-3.29, 0.59) | 0.174 | -1.34 | (-3.29, 0.60) | 0.175 |
| Southeast Asia | 5.96 | (3.51, 8.42) | <0.001 | 5.97 | (3.51, 8.42) | <0.001 |
| Africa | -1.54 | (-3.35, 0.28) | 0.097 | -1.54 | (-3.35, 0.28) | 0.097 |
| Difference-in-difference variables | | | | | | |
| Pre/Post | -2.78 | (-4.86, -0.70) | 0.009 | -3.14 | (-5.36, -0.92) | 0.006 |
| UHC SCI^c^ 2019 ≥80 | -2.86 | (-4.86, -0.86) | 0.005 | -3.01 | (-5.04, -0.98) | 0.004 |
| Pre/Post * UHC SCI^c^ ≥80 |  |  |  | 2.36 | (-2.74, 7.47) | 0.364 |

a: Confidence Interval

b: Global Health Security Index

c: UHC Service Coverage Index

**Table H. Difference-in-difference regression analysis of the third dose of hepatitis B containing vaccine (HEPB3) coverage after COVID-19 pandemic by UHC SCI 2019 (≥80 vs. the rest) - Unadjusted**

|  | Base model | | | Difference-in-difference model | | |
| --- | --- | --- | --- | --- | --- | --- |
| Variable | Coefficient | 95% CI^a^ | p-value | Coefficient | 95% CI^a^ | p-value |
| Intercept | 86.92 | (86.22, 87.61) | <0.001 | 79.47 | (78.41, 80.53) | <0.001 |
| Pre/Post | -3.68 | (-5.97, -1.38) | 0.002 | -2.58 | (-6.19, 1.04) | 0.162 |
| UHC SCI^b^ 2019 ≥80 | 4.05 | (2.31, 5.79) | <0.001 | 11.77 | (10.49, 13.05) | <0.001 |
| Pre/Post * UHC SCI^b^ ≥80 |  |  |  | -1.17 | (-5.65, 3.30) | 0.607 |

a: Confidence Interval

b: UHC Service Coverage Index

**Table I. Difference-in-difference regression analysis of the third dose of hepatitis B containing vaccine (HEPB3) coverage after COVID-19 pandemic by UHC SCI 2019 (≥80 vs. the rest) - Adjusted for calendar year, pandemic preparedness, country income group, and geographic region**

|  | Base model | | | Difference-in-difference model | | |
| --- | --- | --- | --- | --- | --- | --- |
| Variable | Coefficient | 95% CI^a^ | p-value | Coefficient | 95% CI^a^ | p-value |
| Intercept | 134.45 | (-278.08, 546.98) | 0.523 | 133.51 | (-278.85, 545.88) | 0.526 |
| Year | -0.03 | (-0.23, 0.18) | 0.781 | -0.03 | (-0.23, 0.18) | 0.785 |
| GHSI^b^ 2019 | 0.01 | (-0.04, 0.06) | 0.722 | 0.01 | (-0.04, 0.06) | 0.731 |
| World Bank Income Group (Reference category: Low) | | | | | | |
| Lower-middle | 8.11 | (6.18, 10.04) | <0.001 | 8.11 | (6.18, 10.04) | <0.001 |
| Upper-middle | 12.62 | (10.46, 14.78) | <0.001 | 12.61 | (10.45, 14.77) | <0.001 |
| High | 18.04 | (15.59, 20.48) | <0.001 | 18.03 | (15.59, 20.47) | <0.001 |
| WHO Region (Reference category: Americas) | | | | | | |
| Europe | 2.41 | (0.55, 4.26) | 0.011 | 2.4 | (0.54, 4.25) | 0.011 |
| Western Pacific | 1.62 | (-0.46, 3.69) | 0.127 | 1.62 | (-0.46, 3.69) | 0.127 |
| Eastern Mediterranean | -0.33 | (-2.54, 1.88) | 0.768 | -0.32 | (-2.53, 1.88) | 0.775 |
| Southeast Asia | 5.68 | (2.90, 8.46) | <0.001 | 5.68 | (2.90, 8.46) | <0.001 |
| Africa | -0.97 | (-3.04, 1.11) | 0.362 | -0.97 | (-3.04, 1.11) | 0.361 |
| Difference-in-difference variables | | | | | | |
| Pre/Post | -2.96 | (-5.34, -0.59) | 0.015 | -3.65 | (-6.16, -1.13) | 0.005 |
| UHC SCI^c^ 2019 ≥80 | -5.55 | (-7.92, -3.18) | <0.001 | -5.89 | (-8.30, -3.49) | <0.001 |
| Pre/Post * UHC^c^ SCI ≥80 |  |  |  | 5.02 | (-1.11, 11.14) | 0.108 |

a: Confidence Interval

b: Global Health Security Index

c: UHC Service Coverage Index

**Table J. Difference-in-difference regression analysis of the birth dose of hepatitis B containing vaccine (HEPBB) coverage after COVID-19 pandemic by UHC SCI 2019 (≥80 vs. the rest) - Unadjusted**

|  | Base model | | | Difference-in-difference model | | |
| --- | --- | --- | --- | --- | --- | --- |
| Variable | Coefficient | 95% CI^a^ | p-value | Coefficient | 95% CI^a^ | p-value |
| Intercept | 82.16 | (80.59, 83.73) | <0.001 | 78.9 | (75.98, 81.82) | <0.001 |
| Pre/Post | -1.7 | (-6.71, 3.31) | 0.506 | -5.71 | (-14.50, 3.08) | 0.204 |
| UHC SCI^b^ 2019 ≥80 | 7.29 | (2.20, 12.39) | 0.005 | 5.32 | (1.91, 8.72) | 0.002 |
| Pre/Post * UHC SCI^b^ ≥80 |  |  |  | 5.92 | (-4.75, 16.60) | 0.277 |

a: Confidence Interval

b: UHC Service Coverage Index

**Table K. Difference-in-difference regression analysis of the birth dose of hepatitis B containing vaccine (HEPBB) coverage after COVID-19 pandemic by UHC SCI 2019 (≥80 vs. the rest) - Adjusted for calendar year, pandemic preparedness, country income group, and geographic region**

|  | Base model | | | Difference-in-difference model | | |
| --- | --- | --- | --- | --- | --- | --- |
| Variable | Coefficient | 95% CI^a^ | p-value | Coefficient | 95% CI^a^ | p-value |
| Intercept | -543.94 | (-1473.32, 385.43) | 0.252 | -544 | (-1473.95, 385.94) | 0.252 |
| Year | 0.3 | (-0.16, 0.77) | 0.196 | 0.3 | (-0.16, 0.77) | 0.196 |
| GHSI^b^ 2019 | -0.07 | (-0.19, 0.04) | 0.216 | -0.07 | (-0.19, 0.04) | 0.216 |
| World Bank Income Group (Reference category: Low) | | | | | | |
| Lower-middle | -8.33 | (-15.98, -0.67) | 0.033 | -8.33 | (-15.99, -0.67) | 0.033 |
| Upper-middle | 7.54 | (-0.11, 15.20) | 0.054 | 7.54 | (-0.12, 15.20) | 0.054 |
| High | 14.6 | (6.48, 22.72) | <0.001 | 14.6 | (6.47, 22.72) | <0.001 |
| WHO Region (Reference category: Americas) | | | | | | |
| Europe | 22.07 | (18.32, 25.82) | <0.001 | 22.07 | (18.32, 25.82) | <0.001 |
| Western Pacific | 12.25 | (8.23, 16.27) | <0.001 | 12.25 | (8.22, 16.28) | <0.001 |
| Eastern Mediterranean | 6.03 | (1.56, 10.50) | 0.008 | 6.03 | (1.56, 10.51) | 0.008 |
| Southeast Asia | 4.37 | (-1.90, 10.63) | 0.172 | 4.37 | (-1.90, 10.64) | 0.172 |
| Africa | 22.4 | (15.17, 29.62) | <0.001 | 22.39 | (15.17, 29.62) | <0.001 |
| Difference-in-difference variables | | | | | | |
| Pre/Post | -0.27 | (-5.26, 4.71) | 0.915 | -0.26 | (-5.33, 4.80) | 0.919 |
| UHC SCI^c^ 2019 ≥80 | -3.2 | (-8.80, 2.40) | 0.263 | -3.19 | (-8.87, 2.48) | 0.27 |
| Pre/Post * UHC SCI^c^ ≥80 |  |  |  | -0.23 | (-22.42, 21.97) | 0.984 |

a: Confidence Interval

b: Global Health Security Index

c: UHC Service Coverage Index

**Table L. Difference-in-difference regression analysis of the third dose of *Haemophilius influenza* B containing vaccine (HIB3) coverage after COVID-19 pandemic by UHC SCI 2019 (≥80 vs. the rest) - Unadjusted**

|  | Base model | | | Difference-in-difference model | | |
| --- | --- | --- | --- | --- | --- | --- |
| Variable | Coefficient | 95% CI^a^ | p-value | Coefficient | 95% CI^a^ | p-value |
| Intercept | 85.69 | (84.96, 86.41) | <0.001 | 78.31 | (77.19, 79.44) | <0.001 |
| Pre/Post | -3.03 | (-5.39, -0.68) | 0.012 | -1.86 | (-5.65, 1.93) | 0.336 |
| UHC SCI^b^ 2019 ≥80 | 9.35 | (7.70, 10.99) | <0.001 | 13.11 | (11.76, 14.46) | <0.001 |
| Pre/Post * UHC SCI^b^ ≥80 |  |  |  | -1.42 | (-6.10, 3.26) | 0.552 |

a: Confidence Interval

b: UHC Service Coverage Index

**Table M. Difference-in-difference regression analysis of the third dose of *Haemophilius influenza* B containing vaccine (HIB3) coverage after COVID-19 pandemic by UHC SCI 2019 (≥80 vs. the rest) - Adjusted for calendar year, pandemic preparedness, country income group, and geographic region**

|  | Base model | | | Difference-in-difference model | | |
| --- | --- | --- | --- | --- | --- | --- |
| Variable | Coefficient | 95% CI^a^ | p-value | Coefficient | 95% CI^a^ | p-value |
| Intercept | 9.43 | (-424.91, 443.77) | 0.966 | 9.8 | (-424.61, 444.22) | 0.965 |
| Year | 0.03 | (-0.18, 0.25) | 0.769 | 0.03 | (-0.18, 0.25) | 0.77 |
| GHSI^b^ 2019 | 0.08 | (0.03, 0.14) | 0.004 | 0.08 | (0.03, 0.14) | 0.004 |
| World Bank Income Group (Reference category: Low) | | | | | | |
| Lower-middle | 8.3 | (6.26, 10.33) | <0.001 | 8.3 | (6.26, 10.33) | <0.001 |
| Upper-middle | 10.59 | (8.31, 12.88) | <0.001 | 10.59 | (8.31, 12.88) | <0.001 |
| High | 18.5 | (15.96, 21.04) | <0.001 | 18.49 | (15.95, 21.03) | <0.001 |
| WHO Region (Reference category: Americas) | | | | | | |
| Europe | -0.92 | (-2.86, 1.02) | 0.352 | -0.92 | (-2.86, 1.02) | 0.351 |
| Western Pacific | -2.74 | (-4.95, -0.53) | 0.015 | -2.74 | (-4.95, -0.53) | 0.015 |
| Eastern Mediterranean | -2.31 | (-4.66, 0.05) | 0.055 | -2.3 | (-4.65, 0.05) | 0.055 |
| Southeast Asia | 2.38 | (-0.88, 5.65) | 0.153 | 2.39 | (-0.88, 5.66) | 0.152 |
| Africa | -1.65 | (-3.84, 0.53) | 0.139 | -1.65 | (-3.84, 0.53) | 0.139 |
| Difference-in-difference variables | | | | | | |
| Pre/Post | -2.72 | (-5.18, -0.25) | 0.031 | -2.99 | (-5.62, -0.35) | 0.027 |
| UHC SCI^c^ 2019 ≥80 | -1.7 | (-4.08, 0.69) | 0.163 | -1.81 | (-4.22, 0.61) | 0.142 |
| Pre/Post * UHC SCI^c^ ≥80 |  |  |  | 1.74 | (-4.27, 7.76) | 0.57 |

a: Confidence Interval

b: Global Health Security Index

c: UHC Service Coverage Index

**Table N. Difference-in-difference regression analysis of the first dose of measles containing vaccine (MCV1) coverage after COVID-19 pandemic by UHC SCI 2019 (≥80 vs. the rest) - Unadjusted**

|  | Base model | | | Difference-in-difference model | | |
| --- | --- | --- | --- | --- | --- | --- |
| Variable | Coefficient | 95% CI^a^ | p-value | Coefficient | 95% CI^a^ | p-value |
| Intercept | 86.33 | (85.69, 86.97) | <0.001 | 77.69 | (76.76, 78.62) | <0.001 |
| Pre/Post | -3.83 | (-5.94, -1.72) | <0.001 | -3.22 | (-6.43, -0.00) | 0.05 |
| UHC SCI^b^ 2019 ≥80 | 8.13 | (6.67, 9.59) | <0.001 | 14.58 | (13.46, 15.70) | <0.001 |
| Pre/Post * UHC SCI^b^ ≥80 |  |  |  | -0.49 | (-4.45, 3.47) | 0.809 |

a: Confidence Interval

b: UHC Service Coverage Index

**Table O. Difference-in-difference regression analysis of the first dose of measles containing vaccine (MCV1) coverage after COVID-19 pandemic by UHC SCI 2019 (≥80 vs. the rest) - Adjusted for calendar year, pandemic preparedness, country income group, and geographic region**

|  | Base model | | | Difference-in-difference model | | |
| --- | --- | --- | --- | --- | --- | --- |
| Variable | Coefficient | 95% CI^a^ | p-value | Coefficient | 95% CI^a^ | p-value |
| Intercept | 326.24 | (-26.22, 678.70) | 0.07 | 326.33 | (-26.01, 678.66) | 0.07 |
| Year | -0.12 | (-0.30, 0.05) | 0.163 | -0.12 | (-0.30, 0.05) | 0.162 |
| GHSI^b^ 2019 | 0.11 | (0.07, 0.16) | <0.001 | 0.11 | (0.07, 0.16) | <0.001 |
| World Bank Income Group (Reference category: Low) | | | | | | |
| Lower-middle | 8.01 | (6.33, 9.68) | <0.001 | 8.01 | (6.33, 9.68) | <0.001 |
| Upper-middle | 10.71 | (8.86, 12.57) | <0.001 | 10.71 | (8.85, 12.57) | <0.001 |
| High | 16.27 | (14.16, 18.37) | <0.001 | 16.26 | (14.15, 18.36) | <0.001 |
| WHO Region (Reference category: Americas) | | | | | | |
| Europe | 0.38 | (-1.23, 1.98) | 0.646 | 0.37 | (-1.24, 1.98) | 0.651 |
| Western Pacific | -1.51 | (-3.31, 0.29) | 0.1 | -1.51 | (-3.31, 0.29) | 0.101 |
| Eastern Mediterranean | -2.35 | (-4.27, -0.42) | 0.017 | -2.34 | (-4.27, -0.42) | 0.017 |
| Southeast Asia | 3.86 | (1.42, 6.29) | 0.002 | 3.86 | (1.42, 6.29) | 0.002 |
| Africa | -5.6 | (-7.39, -3.80) | <0.001 | -5.6 | (-7.39, -3.80) | <0.001 |
| Difference-in-difference variables | | | | | | |
| Pre/Post | -2.63 | (-4.69, -0.57) | 0.013 | -3.25 | (-5.45, -1.05) | 0.004 |
| UHC SCI^c^ 2019 ≥80 | -3.57 | (-5.56, -1.58) | <0.001 | -3.82 | (-5.84, -1.81) | <0.001 |
| Pre/Post * UHC SCI^c^ ≥80 |  |  |  | 4.05 | (-1.01, 9.10) | 0.117 |

a: Confidence Interval

b: Global Health Security Index

c: UHC Service Coverage Index

**Table P. Difference-in-difference regression analysis of the second dose of measles containing vaccine (MCV2) after COVID-19 pandemic by UHC SCI 2019 (≥80 vs. the rest) - Unadjusted**

|  | Base model | | | Difference-in-difference model | | |
| --- | --- | --- | --- | --- | --- | --- |
| Variable | Coefficient | 95% CI^a^ | p-value | Coefficient | 95% CI^a^ | p-value |
| Intercept | 81.76 | (80.76, 82.76) | <0.001 | 71.65 | (69.80, 73.50) | <0.001 |
| Pre/Post | -5.6 | (-8.62, -2.57) | <0.001 | -8.42 | (-13.74, -3.11) | 0.002 |
| UHC SCI^b^ 2019 ≥80 | 8.59 | (6.48, 10.69) | <0.001 | 15.13 | (13.04, 17.21) | <0.001 |
| Pre/Post * UHC SCI^b^ ≥80 |  |  |  | 5.41 | (-0.92, 11.74) | 0.094 |

a: Confidence Interval

b: UHC Service Coverage Index

**Table Q. Difference-in-difference regression analysis of the second dose of measles containing vaccine (MCV2) after COVID-19 pandemic by UHC SCI 2019 (≥80 vs. the rest) - Adjusted for calendar year, pandemic preparedness, country income group, and geographic region**

|  | Base model | | | Difference-in-difference model | | |
| --- | --- | --- | --- | --- | --- | --- |
| Variable | Coefficient | 95% CI^a^ | p-value | Coefficient | 95% CI^a^ | p-value |
| Intercept | -131 | (-692.48, 430.49) | 0.648 | -126.86 | (-688.39, 434.66) | 0.658 |
| Year | 0.09 | (-0.19, 0.37) | 0.511 | 0.09 | (-0.19, 0.37) | 0.52 |
| GHSI^b^ 2019 | 0.05 | (-0.02, 0.11) | 0.175 | 0.05 | (-0.02, 0.11) | 0.176 |
| World Bank Income Group (Reference category: Low) | | | | | | |
| Lower-middle | 13.09 | (9.89, 16.29) | <0.001 | 13.07 | (9.87, 16.27) | <0.001 |
| Upper-middle | 20.45 | (17.11, 23.78) | <0.001 | 20.41 | (17.08, 23.75) | <0.001 |
| High | 28.01 | (24.47, 31.55) | <0.001 | 27.96 | (24.42, 31.50) | <0.001 |
| WHO Region (Reference category: Americas) | | | | | | |
| Europe | 9.95 | (7.64, 12.26) | <0.001 | 9.93 | (7.62, 12.24) | <0.001 |
| Western Pacific | 3.92 | (1.25, 6.58) | 0.004 | 3.92 | (1.26, 6.58) | 0.004 |
| Eastern Mediterranean | 5.79 | (2.97, 8.61) | <0.001 | 5.79 | (2.97, 8.61) | <0.001 |
| Southeast Asia | 9.06 | (5.42, 12.70) | <0.001 | 9.05 | (5.41, 12.69) | <0.001 |
| Africa | -1.78 | (-4.85, 1.30) | 0.258 | -1.76 | (-4.84, 1.32) | 0.262 |
| Difference-in-difference variables | | | | | | |
| Pre/Post | -2.92 | (-5.93, 0.08) | 0.057 | -3.54 | (-6.76, -0.31) | 0.032 |
| UHC SCI^c^ 2019 ≥80 | -6.28 | (-9.09, -3.47) | <0.001 | -6.53 | (-9.37, -3.68) | <0.001 |
| Pre/Post * UHC SCI^c^ ≥80 |  |  |  | 3.77 | (-3.39, 10.93) | 0.303 |

a: Confidence Interval

b: Global Health Security Index

c: UHC Service Coverage Index

**Table R. Difference-in-difference regression analysis of the third dose of pneumococcal conjugate vaccine (PCV3) coverage after COVID-19 pandemic by UHC SCI 2019 (≥80 vs. the rest) - Unadjusted**

|  | Base model | | | Difference-in-difference model | | |
| --- | --- | --- | --- | --- | --- | --- |
| Variable | Coefficient | 95% CI^a^ | p-value | Coefficient | 95% CI^a^ | p-value |
| Intercept | 78.26 | (76.86, 79.66) | <0.001 | 70.43 | (68.23, 72.62) | <0.001 |
| Pre/Post | 0.64 | (-3.26, 4.54) | 0.748 | 0.67 | (-5.67, 7.00) | 0.837 |
| UHC SCI^b^ 2019 ≥80 | 10.04 | (7.31, 12.77) | <0.001 | 14.56 | (11.95, 17.17) | <0.001 |
| Pre/Post * UHC^b^ SCI ≥80 |  |  |  | 0.52 | (-7.37, 8.41) | 0.897 |

a: Confidence Interval

b: UHC Service Coverage Index

**Table S. Difference-in-difference regression analysis of the third dose of pneumococcal conjugate vaccine (PCV3) coverage after COVID-19 pandemic by UHC SCI 2019 (≥80 vs. the rest) - Adjusted for calendar year, pandemic preparedness, country income group, and geographic region**

|  | Base model | | | Difference-in-difference model | | |
| --- | --- | --- | --- | --- | --- | --- |
| Variable | Coefficient | 95% CI^a^ | p-value | Coefficient | 95% CI^a^ | p-value |
| Intercept | -3226.83 | (-4120.76, -2332.90) | <0.001 | -3233.25 | (-4128.02, -2338.49) | <0.001 |
| Year | 1.63 | (1.19, 2.08) | <0.001 | 1.64 | (1.19, 2.08) | <0.001 |
| GHSI^b^ 2019 | 0.12 | (0.01, 0.23) | 0.04 | 0.12 | (0.00, 0.23) | 0.042 |
| World Bank Income Group (Reference category: Low) | | | | | | |
| Lower-middle | 4.09 | (0.22, 7.96) | 0.038 | 4.09 | (0.22, 7.96) | 0.038 |
| Upper-middle | 6.41 | (1.69, 11.14) | 0.008 | 6.42 | (1.70, 11.15) | 0.008 |
| High | 15.79 | (10.87, 20.72) | <0.001 | 15.81 | (10.88, 20.74) | <0.001 |
| WHO Region (Reference category: Americas) | | | | | | |
| Europe | -6.1 | (-10.41, -1.78) | 0.006 | -6.11 | (-10.42, -1.80) | 0.006 |
| Western Pacific | 3.16 | (-1.20, 7.52) | 0.156 | 3.14 | (-1.22, 7.50) | 0.159 |
| Eastern Mediterranean | -19.85 | (-28.25, -11.45) | <0.001 | -19.91 | (-28.31, -11.51) | <0.001 |
| Southeast Asia | -0.46 | (-4.65, 3.72) | 0.828 | -0.47 | (-4.66, 3.71) | 0.825 |
| Africa | -6.1 | (-10.41, -1.78) | 0.006 | -6.11 | (-10.42, -1.80) | 0.006 |
| Difference-in-difference variables | | | | | | |
| Pre/Post | -5.65 | (-9.84, -1.47) | 0.008 | -5.29 | (-9.81, -0.76) | 0.022 |
| UHC SCI^c^ 2019 ≥80 | -1.04 | (-5.40, 3.32) | 0.64 | -0.88 | (-5.30, 3.55) | 0.698 |
| Pre/Post * UHC SCI^c^ ≥80 |  |  |  | -2 | (-11.41, 7.40) | 0.676 |

a: Confidence Interval

b: Global Health Security Index

c: UHC Service Coverage Index

**Table T. Difference-in-difference regression analysis of the third dose of polio containing vaccine (POL3) coverage after COVID-19 pandemic by UHC SCI 2019 (≥80 vs. the rest) - Unadjusted**

|  | Base model | | | Difference-in-difference model | | |
| --- | --- | --- | --- | --- | --- | --- |
| Variable | Coefficient | 95% CI^a^ | p-value | Coefficient | 95% CI^a^ | p-value |
| Intercept | 86.97 | (86.36, 87.58) | <0.001 | 79.13 | (78.22, 80.03) | <0.001 |
| Pre/Post | -4.27 | (-6.28, -2.25) | <0.001 | -2.34 | (-5.48, 0.79) | 0.143 |
| UHC SCI^b^ 2019 ≥80 | 8.67 | (7.27, 10.07) | <0.001 | 13.57 | (12.48, 14.66) | <0.001 |
| Pre/Post * UHC SCI^b^ ≥80 |  |  |  | -2.57 | (-6.43, 1.29) | 0.192 |

a: Confidence Interval

b: UHC Service Coverage Index

**Table U. Difference-in-difference regression analysis of the third dose of polio containing vaccine (POL3) coverage after COVID-19 pandemic by UHC SCI 2019 (≥80 vs. the rest) - Adjusted for calendar year, pandemic preparedness, country income group, and geographic region**

|  | Base model | | | Difference-in-difference model | | |
| --- | --- | --- | --- | --- | --- | --- |
| Variable | Coefficient | 95% CI^a^ | p-value | Coefficient | 95% CI^a^ | p-value |
| Intercept | 286.06 | (-55.11, 627.24) | 0.1 | 286.13 | (-54.99, 627.26) | 0.1 |
| Year | -0.11 | (-0.27, 0.06) | 0.224 | -0.11 | (-0.27, 0.06) | 0.224 |
| GHSI^b^ 2019 | 0.09 | (0.05, 0.14) | <0.001 | 0.09 | (0.05, 0.14) | <0.001 |
| World Bank Income Group (Reference category: Low) | | | | | | |
| Lower-middle | 7.67 | (6.05, 9.30) | <0.001 | 7.67 | (6.05, 9.30) | <0.001 |
| Upper-middle | 11.3 | (9.50, 13.09) | <0.001 | 11.29 | (9.50, 13.09) | <0.001 |
| High | 16.37 | (14.33, 18.41) | <0.001 | 16.36 | (14.32, 18.40) | <0.001 |
| WHO Region (Reference category: Americas) | | | | | | |
| Europe | 2.65 | (1.09, 4.20) | 0.001 | 2.64 | (1.09, 4.20) | 0.001 |
| Western Pacific | 0.5 | (-1.25, 2.24) | 0.578 | 0.5 | (-1.24, 2.24) | 0.575 |
| Eastern Mediterranean | -0.93 | (-2.80, 0.93) | 0.326 | -0.93 | (-2.79, 0.94) | 0.329 |
| Southeast Asia | 6.16 | (3.80, 8.52) | <0.001 | 6.16 | (3.80, 8.52) | <0.001 |
| Africa | -2.2 | (-3.94, -0.47) | 0.013 | -2.2 | (-3.94, -0.47) | 0.013 |
| Difference-in-difference variables | | | | | | |
| Pre/Post | -3.14 | (-5.13, -1.15) | 0.002 | -3.62 | (-5.75, -1.49) | 0.001 |
| UHC SCI^c^ 2019 ≥80 | -2.6 | (-4.53, -0.68) | 0.008 | -2.8 | (-4.75, -0.85) | 0.005 |
| Pre/Post * UHC SCI^c^ ≥80 |  |  |  | 3.13 | (-1.77, 8.02) | 0.211 |

a: Confidence Interval

b: Global Health Security Index

c: UHC Service Coverage Index

**Table V. Difference-in-difference regression analysis of the second or third dose of rotavirus vaccine (ROTAC) coverage after COVID-19 pandemic by UHC SCI 2019 (≥80 vs. the rest) - Unadjusted**

|  | Base model | | | Difference-in-difference model | | |
| --- | --- | --- | --- | --- | --- | --- |
| Variable | Coefficient | 95% CI^a^ | p-value | Coefficient | 95% CI^a^ | p-value |
| Intercept | 76.22 | (74.39, 78.06) | <0.001 | 70.44 | (67.48, 73.40) | <0.001 |
| Pre/Post | 1.01 | (-3.79, 5.81) | 0.681 | 3.39 | (-4.38, 11.16) | 0.393 |
| UHC SCI^b^ 2019 ≥80 | -0.4 | (-4.52, 3.72) | 0.85 | 8.34 | (4.77, 11.91) | <0.001 |
| Pre/Post * UHC SCI^b^ ≥80 |  |  |  | -2.96 | (-12.77, 6.84) | 0.554 |

a: Confidence Interval

b: UHC Service Coverage Index

**Table W. Difference-in-difference regression analysis of the second or third dose of rotavirus vaccine (ROTAC) coverage after COVID-19 pandemic by UHC SCI 2019 (≥80 vs. the rest) - Adjusted for calendar year, pandemic preparedness, country income group, and geographic region**

|  | Base model | | | Difference-in-difference model | | |
| --- | --- | --- | --- | --- | --- | --- |
| Variable | Coefficient | 95% CI^a^ | p-value | Coefficient | 95% CI^a^ | p-value |
| Intercept | -2544.64 | (-3853.13, -1236.15) | <0.001 | -2543.35 | (-3852.90, -1233.80) | <0.001 |
| Year | 1.3 | (0.65, 1.95) | <0.001 | 1.3 | (0.65, 1.95) | <0.001 |
| GHSI^b^ 2019 | 0.06 | (-0.10, 0.21) | 0.48 | 0.06 | (-0.10, 0.21) | 0.481 |
| World Bank Income Group (Reference category: Low) | | | | | | |
| Lower-middle | 2.87 | (-1.93, 7.66) | 0.242 | 2.87 | (-1.93, 7.67) | 0.242 |
| Upper-middle | 3.54 | (-1.93, 9.00) | 0.205 | 3.53 | (-1.93, 9.00) | 0.206 |
| High | 14.08 | (7.47, 20.69) | <0.001 | 14.08 | (7.46, 20.69) | <0.001 |
| WHO Region (Reference category: Americas) | | | | | | |
| Europe | -13.6 | (-19.07, -8.13) | <0.001 | -13.6 | (-19.07, -8.12) | <0.001 |
| Western Pacific | -13.63 | (-19.88, -7.38) | <0.001 | -13.63 | (-19.88, -7.37) | <0.001 |
| Eastern Mediterranean | -6.02 | (-11.52, -0.52) | 0.032 | -6.01 | (-11.52, -0.50) | 0.033 |
| Southeast Asia | -43.9 | (-61.33, -26.48) | <0.001 | -43.87 | (-61.32, -26.43) | <0.001 |
| Africa | -4.54 | (-9.47, 0.38) | 0.071 | -4.53 | (-9.46, 0.39) | 0.072 |
| Difference-in-difference variables | | | | | | |
| Pre/Post | -3.61 | (-8.97, 1.75) | 0.187 | -3.73 | (-9.50, 2.03) | 0.204 |
| UHC SCI^c^ 2019 ≥80 | -5.94 | (-13.13, 1.25) | 0.106 | -6.03 | (-13.37, 1.32) | 0.108 |
| Pre/Post * UHC SCI^c^ ≥80 |  |  |  | 0.72 | (-11.38, 12.82) | 0.907 |

a: Confidence Interval

b: Global Health Security Index

c: UHC Service Coverage Index

**Table X. Difference-in-difference regression analysis of the first dose of rubella containing vaccine (RCV1) coverage after COVID-19 pandemic by UHC SCI 2019 (**≥**80 vs. the rest) - Unadjusted**

|  | Base model | | | Difference-in-difference model | | |
| --- | --- | --- | --- | --- | --- | --- |
| Variable | Coefficient | 95% CI^a^ | p-value | Coefficient | 95% CI^a^ | p-value |
| Intercept | 90.58 | (89.99, 91.17) | <0.001 | 86.12 | (84.92, 87.32) | <0.001 |
| Pre/Post | -4.46 | (-6.23, -2.69) | <0.001 | -5.31 | (-8.70, -1.92) | 0.002 |
| UHC SCI^b^ 2019 ≥80 | 3.92 | (2.74, 5.10) | <0.001 | 6.57 | (5.24, 7.90) | <0.001 |
| Pre/Post * UHC SCI^b^ ≥80 |  |  |  | 1.66 | (-2.28, 5.61) | 0.409 |

a: Confidence Interval

b: UHC Service Coverage Index

**Table Y. Difference-in-difference regression analysis of the first dose of rubella containing vaccine (RCV1) coverage after COVID-19 pandemic by UHC SCI 2019 (**≥**80 vs. the rest) - Adjusted for calendar year, pandemic preparedness, country income group, and geographic region**

|  | Base model | | | Difference-in-difference model | | |
| --- | --- | --- | --- | --- | --- | --- |
| Variable | Coefficient | 95% CI^a^ | p-value | Coefficient | 95% CI^a^ | p-value |
| Intercept | 569.06 | (214.04, 924.08) | 0.002 | 579.43 | (224.62, 934.25) | 0.001 |
| Year | -0.24 | (-0.42, -0.07) | 0.007 | -0.25 | (-0.42, -0.07) | 0.006 |
| GHSI^b^ 2019 | 0.07 | (0.03, 0.11) | 0.001 | 0.07 | (0.03, 0.11) | 0.001 |
| World Bank Income Group (Reference category: Low) | | | | | | |
| Lower-middle | 6.63 | (4.10, 9.16) | <0.001 | 6.59 | (4.07, 9.12) | <0.001 |
| Upper-middle | 9.92 | (7.38, 12.46) | <0.001 | 9.86 | (7.32, 12.40) | <0.001 |
| High | 13.88 | (11.25, 16.51) | <0.001 | 13.81 | (11.18, 16.43) | <0.001 |
| WHO Region (Reference category: Americas) | | | | | | |
| Europe | 0.74 | (-0.64, 2.12) | 0.293 | 0.73 | (-0.65, 2.11) | 0.299 |
| Western Pacific | -1.38 | (-2.97, 0.20) | 0.087 | -1.37 | (-2.95, 0.21) | 0.089 |
| Eastern Mediterranean | 0.67 | (-1.13, 2.47) | 0.464 | 0.68 | (-1.12, 2.48) | 0.457 |
| Southeast Asia | 1.87 | (-0.59, 4.33) | 0.136 | 1.9 | (-0.56, 4.35) | 0.131 |
| Africa | 1.84 | (-0.25, 3.93) | 0.085 | 1.92 | (-0.17, 4.01) | 0.072 |
| Difference-in-difference variables | | | | | | |
| Pre/Post | -2.21 | (-4.12, -0.30) | 0.024 | -3.01 | (-5.07, -0.95) | 0.004 |
| UHC SCI^c^ 2019 ≥80 | -2.05 | (-3.78, -0.32) | 0.02 | -2.33 | (-4.07, -0.58) | 0.009 |
| Pre/Post * UHC SCI^c^ ≥80 |  |  |  | 4.55 | (0.15, 8.96) | 0.043 |

a: Confidence Interval

b: Global Health Security Index

c: UHC Service Coverage Index

**Table Z. Difference-in-difference regression analysis of overall immunization coverage after COVID-19 pandemic by UHC SCI 2019 (<50 vs. the rest) - Unadjusted**

|  | Base model | | | Difference-in-difference model | | |
| --- | --- | --- | --- | --- | --- | --- |
| Variable | Coefficient | 95% CI^a^ | p-value | Coefficient | 95% CI^a^ | p-value |
| Intercept | 79.52 | (79.15, 79.89) | <0.001 | 79.5 | (79.12, 79.88) | <0.001 |
| Pre/Post | -3.42 | (-4.14, -2.70) | <0.001 | -3.19 | (-4.43, -1.95) | <0.001 |
| UHC SCI^b^ 2019 <50 | 11.47 | (11.03, 11.90) | <0.001 | 11.5 | (11.04, 11.95) | <0.001 |
| Pre/Post * UHC SCI^b^ <50 |  |  |  | -0.35 | (-1.88, 1.17) | 0.651 |

a: Confidence Interval

b: UHC Service Coverage Index

**Table AA. Difference-in-difference regression analysis of overall immunization coverage after COVID-19 pandemic by UHC SCI 2019 (<50 vs. the rest) - Adjusted for calendar year, pandemic preparedness, country income group, geographic region, and vaccine types**

|  | Base model | | | Difference-in-difference model | | |
| --- | --- | --- | --- | --- | --- | --- |
| Variable | Coefficient | 95% CI^a^ | p-value | Coefficient | 95% CI^a^ | p-value |
| Intercept | 38.16 | (-98.22, 174.54) | 0.583 | 37.69 | (-98.70, 174.08) | 0.588 |
| Year | 0.02 | (-0.05, 0.09) | 0.547 | 0.02 | (-0.05, 0.09) | 0.543 |
| GHSI^b^ 2019 | -0.04 | (-0.05, -0.02) | <0.001 | -0.04 | (-0.05, -0.02) | -0.04 |
| World Bank Income Group (Reference category: Low) | | | | | | |
| Lower-middle | 4.83 | (4.15, 5.51) | <0.001 | 4.83 | (4.15, 5.51) | <0.001 |
| Upper-middle | 8.03 | (7.26, 8.80) | <0.001 | 8.03 | (7.26, 8.80) | <0.001 |
| High | 11.68 | (10.85, 12.51) | <0.001 | 11.68 | (10.84, 12.51) | <0.001 |
| WHO Region (Reference category: Americas) | | | | | | |
| Europe | 2.81 | (2.22, 3.39) | <0.001 | 2.81 | (2.22, 3.39) | <0.001 |
| Western Pacific | 2.72 | (2.02, 3.42) | <0.001 | 2.72 | (2.02, 3.43) | <0.001 |
| Eastern Mediterranean | -0.77 | (-1.49, -0.06) | 0.034 | -0.78 | (-1.49, -0.06) | 0.034 |
| Southeast Asia | 5.43 | (4.47, 6.38) | <0.001 | 5.43 | (4.47, 6.38) | <0.001 |
| Africa | 0.27 | (-0.43, 0.96) | 0.452 | 0.26 | (-0.43, 0.96) | 0.454 |
| Vaccine type (Reference category: BCG^o^) | | | | | | |
| DTP1^c^ | 0.44 | (-0.42, 1.29) | 0.316 | 0.44 | (-0.42, 1.29) | 0.316 |
| DTP3^d^ | -4 | (-4.86, -3.15) | <0.001 | -4 | (-4.86, -3.15) | <0.001 |
| HEPB3^e^ | -4.79 | (-5.66, -3.93) | <0.001 | -4.79 | (-5.66, -3.93) | <0.001 |
| HEPBB^f^ | -11.03 | (-12.14, -9.93) | <0.001 | -11.04 | (-12.14, -9.93) | <0.001 |
| HIB3^g^ | -5.1 | (-5.97, -4.24) | <0.001 | -5.1 | (-5.97, -4.24) | <0.001 |
| MCV1^h^ | -4.79 | (-5.64, -3.93) | <0.001 | -4.79 | (-5.64, -3.93) | <0.001 |
| MCV2^i^ | -11.02 | (-11.93, -10.10) | <0.001 | -11.02 | (-11.93, -10.11) | <0.001 |
| PCV3^j^ | -11.5 | (-12.50, -10.49) | <0.001 | -11.5 | (-12.50, -10.50) | <0.001 |
| POL3^k^ | -4.08 | (-4.94, -3.23) | <0.001 | -4.08 | (-4.94, -3.23) | <0.001 |
| RCV1^l^ | -3.56 | (-4.48, -2.64) | <0.001 | -3.57 | (-4.49, -2.64) | <0.001 |
| ROTAC^m^ | -14.68 | (-15.84, -13.53) | <0.001 | -14.69 | (-15.84, -13.53) | <0.001 |
| Difference-in-difference variables | | | | | | |
| Pre/Post | -2.65 | (-3.41, -1.89) | <0.001 | -2.28 | (-3.48, -1.08) | <0.001 |
| UHC SCI^n^ 2019 <50 | 7.61 | (7.08, 8.15) | <0.001 | 7.67 | (7.11, 8.22) | <0.001 |
| Pre/Post * UHC^n^ SCI <50 |  |  |  | -0.56 | (-1.98, 0.86) | 0.438 |

a: Confidence Interval

b: Global Health Security Index

c: diphtheria, tetanus toxoid, and pertussis containing vaccine – first dose

d: diphtheria, tetanus toxoid, and pertussis containing vaccine – third dose

e: hepatitis B vaccine – third dose

f: hepatitis B vaccine – birth dose

g: *Haemophilus influenzae* type B containing vaccine

h: measles containing vaccine – first dose

i: measles containing vaccine – third dose

j: pneumococcal conjugate vaccine – third dose

k: polio containing vaccine – third dose

l: rubella containing vaccine – first dose

m: rotavirus vaccine – second or third dose

n: UHC Service Coverage Index

o: Bacille Calmette-Guérin
