# Supplementary material for: Universal healthcare coverage and health service delivery before and during the COVID-19 pandemic: A difference-in-difference study of childhood immunization coverage from 195 countries
Source: PLoS Med. 2022 Aug 16;19(8):e1004060. doi: 10.1371/journal.pmed.1004060 (PMC9380914; doi:10.1371/journal.pmed.1004060)
Supplement: S3 File — Fig A. Adjusted difference-in-difference coefficient from the analysis replicated with a range of cutoff values threshold (50–80) for UHC SCI 2019. Table A. Adjusted difference-in-difference coefficient from the analysis replicated with a range of cutoff values threshold (50–80) for UHC SCI 2019. Table B. Difference-in-difference regression analysis of overall immunization coverage after COVID-19 pandemic by UHC SCI 2019 (> = 80 vs. the rest) from 2015 to 2020—Unadjusted. Table C. Difference-in-difference regression analysis of overall immunization coverage after COVID-19 pandemic by UHC SCI 2019 (> = 80 vs. the rest) from 2015 to 2020—Adjusted for calendar year, pandemic preparedness, country income group, geographic region, and vaccine types. Table D. Difference-in-difference regression analysis of overall immunization coverage after COVID-19 pandemic by UHC SCI 2019 (<50 vs. the rest) from 2015 to 2020—Unadjusted. Table E. Difference-in-difference regression analysis of overall immunization coverage after COVID-19 pandemic by UHC SCI 2019 (<50 vs. the rest) from 2015 to 2020—Adjusted for calendar year, pandemic preparedness, country income group, geographic region, and vaccine types. (DOCX) [file pmed.1004060.s004.docx]

**Universal Healthcare Coverage and Health Service Delivery in Times of Public Health Crises: A Difference-in-Difference Study of Childhood Immunization Coverage from 195 Countries Before and During the COVID-19 Pandemic**

**Supporting Information 3**


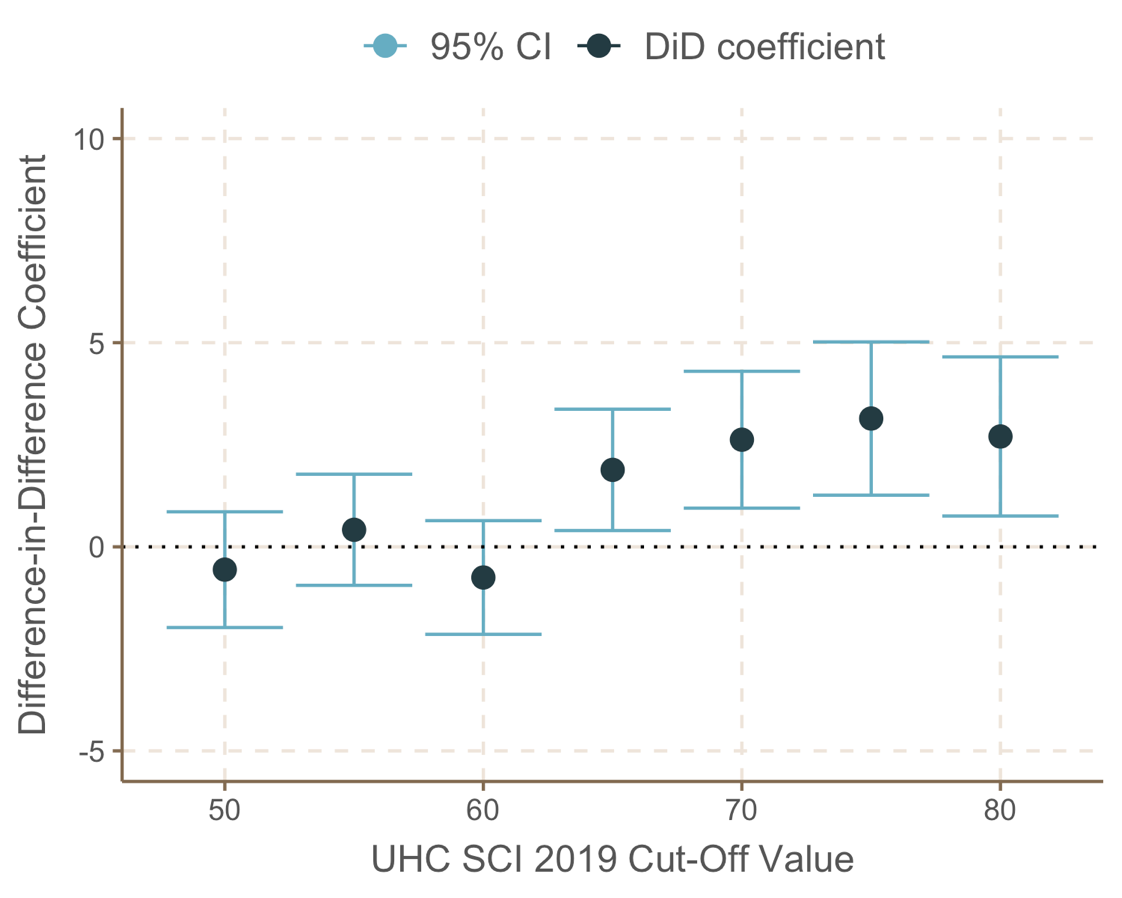


**Figure A. Adjusted difference-in-difference coefficient from the analysis replicated with a range of cutoff values threshold (50-80) for UHC SCI 2019**

**Table A.** **Adjusted difference-in-difference coefficient from the analysis replicated with a range of cutoff values threshold (50-80) for UHC SCI 2019**

| UHC SCI 2019 cutoff threshold | Difference-in-difference coefficient | 95% confidence interval | p-value |
| --- | --- | --- | --- |
| 50 | -0.561 | (-1.978, 0.856) | 0.4378 |
| 55 | 0.416 | (-0.945, 1.778) | 0.5491 |
| 60 | -0.753 | (-2.145, 0.640) | 0.2894 |
| 65 | 1.884 | (0.397, 3.371) | 0.013 |
| 70 | 2.623 | (0.947, 4.299) | 0.0022 |
| 75 | 3.142 | (1.264, 5.020) | 0.001 |
| 80 | 2.702 | (0.754, 4.650) | 0.0066 |

**Replicating the analyses using the shortened pre-period (2015 – 2019)**

**Table B. Difference-in-difference regression analysis of overall immunization coverage after COVID-19 pandemic by UHC SCI 2019 (>= 80 vs. the rest) from 2015 to 2020 – Unadjusted**

|  | Base model | | | Difference-in-difference model | | |
| --- | --- | --- | --- | --- | --- | --- |
| Variable | Coefficient | 95% CI^a^ | p-value | Coefficient | 95% CI^a^ | p-value |
| Intercept | 86.04 | (85.71, 86.36) | <0.001 | 86.1 | (85.78, 86.43) | <0.001 |
| Pre/Post | -3.38 | (-4.16, -2.59) | <0.001 | -3.82 | (-4.67, -2.98) | <0.001 |
| UHC SCI^b^ 2019 >=80 | 6.96 | (6.22, 7.70) | <0.001 | 6.57 | (5.77, 7.36) | <0.001 |
| Pre/Post * UHC SCI^b^ >=80 |  |  |  | 3.11 | (0.88, 5.34) | 0.006 |

a: Confidence Interval

b: UHC Service Coverage Index

**Table C. Difference-in-difference regression analysis of overall immunization coverage after COVID-19 pandemic by UHC SCI 2019 (>= 80 vs. the rest) from 2015 to 2020 - Adjusted for calendar year, pandemic preparedness, country income group, geographic region, and vaccine types**

|  | Base model | | | Difference-in-difference model | | |
| --- | --- | --- | --- | --- | --- | --- |
| Variable | Coefficient | 95% CI^a^ | p-value | Coefficient | 95% CI^a^ | p-value |
| Intercept | -74.76 | (-461.04, 311.53) | 0.704 | -74 | (-460.21, 312.21) | 0.707 |
| Year | 0.08 | (-0.12, 0.27) | 0.438 | 0.08 | (-0.12, 0.27) | 0.44 |
| GHSI^b^ 2019 | 0.07 | (0.05, 0.10) | <0.001 | 0.07 | (0.05, 0.10) | <0.001 |
| World Bank Income Group (Reference category: Low) | | | | | | |
| Lower-middle | 6.93 | (6.06, 7.81) | <0.001 | 6.93 | (6.06, 7.81) | <0.001 |
| Upper-middle | 10.85 | (9.88, 11.83) | <0.001 | 10.85 | (9.87, 11.82) | <0.001 |
| High | 17.03 | (15.94, 18.13) | <0.001 | 17.02 | (15.93, 18.12) | <0.001 |
| WHO Region (Reference category: Americas) | | | | | | |
| Europe | 2.87 | (2.05, 3.69) | <0.001 | 2.86 | (2.04, 3.68) | <0.001 |
| Western Pacific | 0.64 | (-0.28, 1.56) | 0.173 | 0.64 | (-0.27, 1.56) | 0.169 |
| Eastern Mediterranean | 0.25 | (-0.72, 1.23) | 0.609 | 0.26 | (-0.71, 1.23) | 0.599 |
| Southeast Asia | 5.07 | (3.80, 6.34) | <0.001 | 5.07 | (3.81, 6.34) | <0.001 |
| Africa | -0.02 | (-0.95, 0.90) | 0.966 | -0.02 | (-0.94, 0.91) | 0.969 |
| Vaccine type (Reference category: BCG^o^) | | | | | | |
| DTP1^c^ | 0.81 | (-0.38, 2.00) | 0.182 | 0.81 | (-0.38, 2.00) | 0.183 |
| DTP3^d^ | -3.69 | (-4.89, -2.50) | <0.001 | -3.7 | (-4.89, -2.50) | <0.001 |
| HEPB3^e^ | -4.31 | (-5.51, -3.11) | <0.001 | -4.32 | (-5.52, -3.12) | <0.001 |
| HEPBB^f^ | -10.33 | (-11.81, -8.84) | <0.001 | -10.31 | (-11.80, -8.83) | <0.001 |
| HIB3^g^ | -4.42 | (-5.61, -3.22) | <0.001 | -4.42 | (-5.61, -3.22) | <0.001 |
| MCV1^h^ | -4.57 | (-5.76, -3.38) | <0.001 | -4.57 | (-5.77, -3.38) | <0.001 |
| MCV2^i^ | -10.51 | (-11.75, -9.28) | <0.001 | -10.51 | (-11.75, -9.27) | <0.001 |
| PCV3^j^ | -8.98 | (-10.29, -7.68) | <0.001 | -8.99 | (-10.29, -7.68) | <0.001 |
| POL3^k^ | -3.78 | (-4.98, -2.59) | <0.001 | -3.78 | (-4.98, -2.59) | <0.001 |
| RCV1^l^ | -3.07 | (-4.32, -1.82) | <0.001 | -3.07 | (-4.32, -1.82) | <0.001 |
| ROTAC^m^ | -12.06 | (-13.51, -10.61) | <0.001 | -12.07 | (-13.52, -10.62) | <0.001 |
| Difference-in-difference variables | | | | | | |
| Pre/Post | -2.9 | (-3.81, -1.99) | <0.001 | -3.25 | (-4.21, -2.30) | <0.001 |
| UHC SCI^n^ 2019 >=80 | -3.76 | (-4.80, -2.72) | <0.001 | -4.05 | (-5.11, -2.98) | <0.001 |
| Pre/Post * UHC SCI^n^ >=80 |  |  |  | 2.41 | (0.39, 4.42) | 0.019 |

a: Confidence Interval

b: Global Health Security Index

c: diphtheria, tetanus toxoid, and pertussis containing vaccine – first dose

d: diphtheria, tetanus toxoid, and pertussis containing vaccine – third dose

e: hepatitis B vaccine – third dose

f: hepatitis B vaccine – birth dose

g: *Haemophilus influenzae* type B containing vaccine

h: measles containing vaccine – first dose

i: measles containing vaccine – third dose

j: pneumococcal conjugate vaccine – third dose

k: polio containing vaccine – third dose

l: rubella containing vaccine – first dose

m: rotavirus vaccine – second or third dose

n: UHC Service Coverage Index

o: Bacille Calmette-Guérin

**Table D. Difference-in-difference regression analysis of overall immunization coverage after COVID-19 pandemic by UHC SCI 2019 (< 50 vs. the rest) from 2015 to 2020 – Unadjusted**

|  | Base model | | | Difference-in-difference model | | |
| --- | --- | --- | --- | --- | --- | --- |
| Variable | Coefficient | 95% CI^a^ | p-value | Coefficient | 95% CI^a^ | p-value |
| Intercept | 79.2 | (78.71, 79.68) | <0.001 | 79.15 | (78.64, 79.67) | <0.001 |
| Pre/Post^b^ | -3.11 | (-3.86, -2.37) | <0.001 | -2.84 | (-4.12, -1.56) | <0.001 |
| UHC SCI^c^ 2019 <50 | 11.5 | (10.93, 12.06) | <0.001 | 11.56 | (10.94, 12.18) | <0.001 |
| Pre/Post * UHC SCI^c^ <50 |  |  |  | -0.41 | (-1.99, 1.16) | 0.607 |

a: Confidence Interval

b: Pre period refers to the years 2015-2019 and the Post period refers to the year 2020

c: UHC Service Coverage Index

**Table E. Difference-in-difference regression analysis of overall immunization coverage after COVID-19 pandemic by UHC SCI 2019 (< 50 vs. the rest) from 2015 to 2020 - Adjusted for calendar year, pandemic preparedness, country income group, geographic region, and vaccine types**

|  | Base model | | | Difference-in-difference model | | |
| --- | --- | --- | --- | --- | --- | --- |
| Variable | Coefficient | 95% CI^a^ | p-value | Coefficient | 95% CI^a^ | p-value |
| Intercept | -96.67 | (-476.30, 282.96) | 0.618 | -96.99 | (-476.64, 282.66) | 0.617 |
| Year | 0.09 | (-0.10, 0.28) | 0.365 | 0.09 | (-0.10, 0.28) | 0.364 |
| GHSI 2019 | -0.02 | (-0.05, -0.00) | 0.042 | -0.02 | (-0.05, -0.00) | 0.041 |
| World Bank Income Group (Reference category: Low) | | | | | | |
| Lower-middle | 4.64 | (3.75, 5.53) | <0.001 | 4.64 | (3.75, 5.53) | <0.001 |
| Upper-middle | 7.38 | (6.36, 8.39) | <0.001 | 7.38 | (6.36, 8.39) | <0.001 |
| High | 11.59 | (10.50, 12.69) | <0.001 | 11.59 | (10.49, 12.69) | <0.001 |
| WHO Region (Reference category: Americas) | | | | | | |
| Europe | 3.07 | (2.29, 3.86) | <0.001 | 3.07 | (2.28, 3.86) | <0.001 |
| Western Pacific | 3.35 | (2.40, 4.29) | <0.001 | 3.35 | (2.41, 4.29) | <0.001 |
| Eastern Mediterranean | -0.12 | (-1.08, 0.83) | 0.8 | -0.13 | (-1.08, 0.83) | 0.796 |
| Southeast Asia | 6.81 | (5.55, 8.06) | <0.001 | 6.8 | (5.55, 8.06) | <0.001 |
| Africa | 0.96 | (0.04, 1.87) | 0.04 | 0.95 | (0.04, 1.87) | 0.041 |
| Vaccine type (Reference category: BCG^o^) | | | | | | |
| DTP1^c^ | 0.65 | (-0.52, 1.82) | 0.273 | 0.65 | (-0.52, 1.82) | 0.273 |
| DTP3^d^ | -3.85 | (-5.02, -2.68) | <0.001 | -3.85 | (-5.02, -2.68) | <0.001 |
| HEPB3^e^ | -4.46 | (-5.64, -3.28) | <0.001 | -4.46 | (-5.64, -3.28) | <0.001 |
| HEPBB^f^ | -10.29 | (-11.75, -8.83) | <0.001 | -10.29 | (-11.76, -8.83) | <0.001 |
| HIB3^g^ | -4.56 | (-5.73, -3.38) | <0.001 | -4.56 | (-5.73, -3.38) | <0.001 |
| MCV1^h^ | -4.73 | (-5.90, -3.56) | <0.001 | -4.73 | (-5.90, -3.56) | <0.001 |
| MCV2^i^ | -10.82 | (-12.04, -9.61) | <0.001 | -10.83 | (-12.04, -9.61) | <0.001 |
| PCV3^j^ | -8.94 | (-10.22, -7.65) | <0.001 | -8.94 | (-10.22, -7.65) | <0.001 |
| POL3^k^ | -3.94 | (-5.11, -2.77) | <0.001 | -3.94 | (-5.11, -2.77) | <0.001 |
| RCV1^l^ | -3.41 | (-4.64, -2.18) | <0.001 | -3.41 | (-4.64, -2.18) | <0.001 |
| ROTAC^m^ | -12.02 | (-13.45, -10.60) | <0.001 | -12.02 | (-13.45, -10.60) | <0.001 |
| Difference-in-difference variables | | | | | | |
| Pre/Post | -2.85 | (-3.74, -1.95) | <0.001 | -2.63 | (-3.95, -1.31) | <0.001 |
| UHC SCI^n^ 2019 <50 | 7.67 | (6.96, 8.38) | <0.001 | 7.72 | (6.98, 8.47) | <0.001 |
| Pre/Post * UHC SCI^n^ <50 |  |  |  | -0.34 | (-1.81, 1.14) | 0.655 |

a: Confidence Interval

b: Global Health Security Index

c: diphtheria, tetanus toxoid, and pertussis containing vaccine – first dose

d: diphtheria, tetanus toxoid, and pertussis containing vaccine – third dose

e: hepatitis B vaccine – birth dose

f: hepatitis B vaccine – third dose

g: *Haemophilus influenzae* type B containing vaccine

h: measles containing vaccine – first dose

i: measles containing vaccine – third dose

j: pneumococcal conjugate vaccine – third dose

k: polio containing vaccine – third dose

l: rubella containing vaccine – first dose

m: rotavirus vaccine – second or third dose

n: UHC Service Coverage Index

o: Bacille Calmette-Guérin
