# Supplementary material for: Universal healthcare coverage and health service delivery before and during the COVID-19 pandemic: A difference-in-difference study of childhood immunization coverage from 195 countries
Source: PLoS Med. 2022 Aug 16;19(8):e1004060. doi: 10.1371/journal.pmed.1004060 (PMC9380914; doi:10.1371/journal.pmed.1004060)
Supplement: S1 STROBE Checklist — Table A. STROBE Statement—Checklist of items that should be included in reports of cross-sectional studies. (DOCX) [file pmed.1004060.s005.docx]

A. STROBE Statement—Checklist of items that should be included in reports of ***cross-sectional studies***

|  | Item No | Recommendation | Page No |
| --- | --- | --- | --- |
| **Title and abstract** | 1 | (*a*) Indicate the study’s design with a commonly used term in the title or the abstract | Title; Abstract>Methods section |
|  |  | (*b*) Provide in the abstract an informative and balanced summary of what was done and what was found | Abstract>Methods and Results section |
| Introduction | | | |
| Background/rationale | 2 | Explain the scientific background and rationale for the investigation being reported | Introduction paragraphs 1-5 |
| Objectives | 3 | State specific objectives, including any prespecified hypotheses | Introduction paragraph 4 |
| Methods | | | |
| Study design | 4 | Present key elements of study design early in the paper | Methods>Data analysis section paragraph 1-2 |
| Setting | 5 | Describe the setting, locations, and relevant dates, including periods of recruitment, exposure, follow-up, and data collection | Methods>Data section |
| Participants | 6 | (*a*) Give the eligibility criteria, and the sources and methods of selection of participants | Methods>Data section |
| Variables | 7 | Clearly define all outcomes, exposures, predictors, potential confounders, and effect modifiers. Give diagnostic criteria, if applicable | Methods>Data analysis section paragraph 3-5 |
| Data sources/ measurement | 8* | For each variable of interest, give sources of data and details of methods of assessment (measurement). Describe comparability of assessment methods if there is more than one group | Methods>Data section; Supporting Information 1 (S1) |
| Bias | 9 | Describe any efforts to address potential sources of bias | Methods>Data analysis section paragraph 6-10 |
| Study size | 10 | Explain how the study size was arrived at | Methods> Data section; Table 1; Supporting Information Tables S1-1,3 and 4 |
| Quantitative variables | 11 | Explain how quantitative variables were handled in the analyses. If applicable, describe which groupings were chosen and why | Methods>Data analysis section paragraph 3-5 |
| Statistical methods | 12 | (*a*) Describe all statistical methods, including those used to control for confounding | Methods>Data analysis section paragraph 6-10 |
|  |  | (*b*) Describe any methods used to examine subgroups and interactions | Methods>Data analysis section paragraphs 6-11 |
|  |  | (*c*) Explain how missing data were addressed | Methods>Data analysis section 5 |
|  |  | (*d*) If applicable, describe analytical methods taking account of sampling strategy | N/A |
|  |  | (*e*) Describe any sensitivity analyses | Methods>Data analysis section paragraph 11 |
| Results | | | |
| Participants | 13* | (a) Report numbers of individuals at each stage of study—eg numbers potentially eligible, examined for eligibility, confirmed eligible, included in the study, completing follow-up, and analysed | Results paragraph 1,2; Table 1; Supporting Information Tables S1-1,3 and 4 |
|  |  | (b) Give reasons for non-participation at each stage | Results paragraph 1,2 |
|  |  | (c) Consider use of a flow diagram | N/A |
| Descriptive data | 14* | (a) Give characteristics of study participants (eg demographic, clinical, social) and information on exposures and potential confounders | Results paragraph 1,2; Table 1; Supporting Information Tables S1-1,3 and 4 |
|  |  | (b) Indicate number of participants with missing data for each variable of interest | Supporting Information Tables S1-3 and 4 |
| Outcome data | 15* | Report numbers of outcome events or summary measures | Table 2 and 3, Results paragraph 3-5 |
| Main results | 16 | (*a*) Give unadjusted estimates and, if applicable, confounder-adjusted estimates and their precision (eg, 95% confidence interval). Make clear which confounders were adjusted for and why they were included | Table 2 and 3, Supporting Information Tables 2-1 ~ 24 |
|  |  | (*b*) Report category boundaries when continuous variables were categorized | Table 2 and 3, Supporting Information Tables 2-1 ~ 24 |
|  |  | (*c*) If relevant, consider translating estimates of relative risk into absolute risk for a meaningful time period | N/A |
| Other analyses | 17 | Report other analyses done—eg analyses of subgroups and interactions, and sensitivity analyses | Supporting Information Tables 3-1~4, Figure 3-1, Results paragraph 4 |
| Discussion | | | |
| Key results | 18 | Summarise key results with reference to study objectives | Discussion paragraphs 1-3 |
| Limitations | 19 | Discuss limitations of the study, taking into account sources of potential bias or imprecision. Discuss both direction and magnitude of any potential bias | Discussion paragraphs 4-8 |
| Interpretation | 20 | Give a cautious overall interpretation of results considering objectives, limitations, multiplicity of analyses, results from similar studies, and other relevant evidence | Discussion paragraph 2, 3, 9 |
| Generalisability | 21 | Discuss the generalisability (external validity) of the study results | Discussion paragraph 2, 3, 9 |
| Other information | | | |
| Funding | 22 | Give the source of funding and the role of the funders for the present study and, if applicable, for the original study on which the present article is based | N/A (not funded) |

*Give information separately for exposed and unexposed groups.

**Note:** An Explanation and Elaboration article discusses each checklist item and gives methodological background and published examples of transparent reporting. The STROBE checklist is best used in conjunction with this article (freely available on the Web sites of PLoS Medicine at http://www.plosmedicine.org/, Annals of Internal Medicine at http://www.annals.org/, and Epidemiology at http://www.epidem.com/). Information on the STROBE Initiative is available at www.strobe-statement.org.
