# Supplementary material for: Universal healthcare coverage and health service delivery before and during the COVID-19 pandemic: A difference-in-difference study of childhood immunization coverage from 195 countries
Source: PLoS Med. 2022 Aug 16;19(8):e1004060. doi: 10.1371/journal.pmed.1004060 (PMC9380914; doi:10.1371/journal.pmed.1004060)
Supplement: Attachment — Submitted filename: (Final) Response to editors and reviewers.docx [file pmed.1004060.s006.docx]

**Response to Reviewers (Manuscript PMEDICINE-D-22-00227R1)**

Dear editor and reviewers,

On behalf of my co-authors, I would like to thank you for your constructive comments on our manuscript. Please find below an itemized list of our responses.

In summary, we 1) reinforced our Methods section by providing more information on the data used in the analyses, 2) added several analyses to ensure our findings are robust, and 3) toned down the overall language interpreting our findings to avoid asserting causality and to allow for alternative explanations.

We followed your guidance and updated our manuscript to meet PLOS Medicine’s requirements, to include updating our data availability statement. We additionally confirm that this material has not and will not be submitted to any other journal for publication so long as this manuscript is under consideration at PLOS Medicine.

Please do not hesitate to reach out to me with questions.

Sincerely,

Yesim Tozan, PhD

Comments from the reviewers:

**Reviewer #1:** This paper provides a clearly written account of an effort to understand the relationship between universal healthcare coverage and declines in immunization coverage during the pandemic. Overall, the topic is important and the logic of the paper is sound. However, some details, which would be helpful to the reader, are omitted (I've outlined these below).

1.    Abstract: This sentence is unclear: "The DiD estimators indicated that countries with a high UHC index (UHC SCI380) prevented a 2.93% reduction in childhood immunization coverage during the pandemic year of 2020 as compared to the control group (p-value=0.01)." I think my confusion comes from the mixing of causal and descriptive language. Did having high UHC prevent a 2.93% reduction or was the reduction among countries with high UHC 2.93% less than the reduction among countries with low UHC?

-> Thank you for pointing this out. We updated the sentence to “The DiD estimators indicated that countries with a high UHC index (UHC SCI>=80) had a 2.93% less reduction in childhood immunization coverage during the pandemic year of 2020 as compared to the control group.” Per your comment, we believe that this sentence more clearly lays out the relationship.

2.    The paper models immunization coverage (which is a proportion) as a function of covariates and DiD variables using linear regression. Given that immunization coverage for some vaccines is near 1 in some groups, linear regression may not be appropriate (that is, it would be easy to obtain predictive values above 1). Some justification for the use of a linear model should be included here.

-> Thank you for this comment. We chose linear regression as our analytical approach given the continuous outcome variable and the observed linear trend of the outcome variable during the pre-period (2010-2019). To better explain the rationale and to be transparent about its limitation, we added our justification on the choice of regression methods in Lines 247-253.

3.    Did the model perform any type of weighting for population?

-> Thank you for this question. We did not account for the population size based on the assumption that country’s population size is not a direct confounder in the association between immunization coverage and the interaction between the COVID-19 shock and country’s UHC index. A review of the literature indicated that immunization coverage may be associated with the relative degree of health services provision to population, such as health worker density per population or the size of catchment area per health center rather than the population size per se (See: <https://human-resources-health.biomedcentral.com/articles/10.1186/1478-4491-6-29> or <https://academic.oup.com/ije/article-abstract/22/6/1146/650231>). Further, this relative measure of service provision/coverage is somewhat incorporated in the UHC index. We added additional language (Lines 148-155), which we hope clarifies our rationale.

4.    Page 9, last few sentences: how were covariates included in the model (e.g., linear terms, splines, indicator variables?) and how were functional forms chosen?

-> Thank you for your question. Per your comment, we expounded on the covariates included in the model and how the functional forms were chosen (Lines 155-167 and Lines 242-246).

5.    Page 9, last sentence: how was pandemic preparedness operationalized? Specifically, was UHC part of this definition?

-> We incorporated the Global Health Security Index to help operationalize pandemic preparedness. We have added additional clarifying language in our Methods section (Lines 156-163) and we modified a sentence on Page 9 (Lines 242-246) to make this more clear.

6.    Page 12, lines 2-4: was the -2.94% computed from the model without the interaction term? This analysis should be described in the methods section. Moreover, I would change "statistically significant and negative" to "lower", or simply use the second part of this sentence that is easier to understand.

-> Thank you for this comment. We simplified the sentence to make the interpretation more clear to the readers (Lines 290-296), and took your suggestion to change “statistically significant and negative” to “lower.” The methods and the results sections now include a description of base model (Lines 227-234, Tables 2).

7.    How was the summary immunization coverage metric computed (both overall and for individual vaccines? Some details should be included in the Methods. (I see that the text references the WHO/UNICEF Joint Estimates. It would suffice here to give a rough overview of what the immunization coverage metric is and how to interpret it).

-> Thank you for this comment. We added a detailed specification of all the metrics used in the analysis and also included citations to the papers describing the WHO/UNICEF Joint Estimates’ calculation methods in detail (Lines 143-146).

8.    The paper used the UHC SCI estimates from 2019. Is there any evidence that the pandemic affected UHC? If so, what are the implications for the results?

-> Thank you for providing this insight. We searched PubMed with no restrictions on language or time of publication, using the following search terms: ((UHC or "Universal health coverage") AND (crisis OR crises OR emergency OR pandemic OR epidemic) AND (resilience OR protect* OR impact OR affect OR disrupt*)). The search yielded 534 articles. After screening titles and abstracts, we identified 15 articles that discussed the country’s UHC and health system performance in times of public health crises. All were qualitative studies, and all but one focused on the COVID-19 pandemic. All studies suggested that UHC was important for building health system resilience against the COVID-19 pandemic, none suggested any evidence of the pandemic’s disruptive effect on UHC. We thus can’t yet postulate on the potential effects of the pandemic on UHC but highlight that this could be an area for future research.

9.    How were specific vaccines that are not typically administered in one or more countries handled? (e.g., BCG in the US)

-> The main analysis includes all data points available for country-vaccine pairs. If the country doesn’t administer a specific vaccine, they’re excluded from the vaccine type-specific analysis.

**Reviewer #2:** The authors describe an analysis of the influence of universal health coverage (UHC) on the level of disruption to childhood immunisation due to the COVID-19 pandemic. They used a difference-in-difference approach to examine the relative change for each country given their achieved UHC. They found a higher UHC had a protective effect for routine immunisation services in the pandemic. The paper was generally very well written, made some strong points, and I enjoyed reading it.

I have some comments on improvements:

1. The threshold for the UHC cutoff feels a little arbitrary and I think a small amount of work would help justify this (even with the sensitivity analysis at UHC=50). For example, Figure S1-2 would suggest a cutoff of 75 would split the distributions into two more distinct groups. You could perform a Kolmogorov-Smirnov test on a range of cutoff values to find the most distinct split. In this way, the threshold itself may also be informative.

-> Thank you for this note. While we agree that a cutoff value of 75 may split the distributions into more distinct groups, we note that the cutoff’s purpose was not to bifurcate the empirical score distribution into two most distinctive groups, but rather to define what level of UHC index qualifies as sufficiently high coverage. Therefore, even if the distribution splits better at 75, we used a cutoff value of 80 for our main results section given that prior literature indicated that this may qualify as sufficiently high. Based on your suggestion, we also investigated the Kolmogorov-Smirnov test. It seems that Kolmogorov-Smirnov test is used when determining the cutoff of binary classification based on the predicted continuous probability. To apply this in our context, the UHC index needs to be predicted using a set of predictors so that we can compare the predicted score distribution to the empirical distribution of UHC index using the Kolmogorov-Smirnov test. With the lack of the predictors to conduct the first step, we unfortunately couldn’t apply the test. Alternatively, we replicated the same analysis using the sliding scale of cutoff values between 50 and 80, with step increments of 5, to evaluate the threshold of UHC SCI 2019 where significant resilience occur. We included this finding in the Supporting Information (Figure S3-1) and described in the methods and our interpretation in the main manuscript (Lines 258-260, Lines 306-310), which we believe helps address your note about more distinct groups.

2. It would be worth discussing the other indicators used in UHC to highlight the value added by this analysis and to illustrate that high UHC does not necessarily only depend on high immunisation rates. Otherwise, the argument may appear circular.

-> Thank you for this important suggestion. We added sentences summarizing the framework and measurement of UHC SCI 2019 in the Methods (Lines 146-155) to clearly illustrate that UHC is not solely dependent on high immunization rates.

3. Whilst vaccination coverage may be easily accessible it is subject to large uncertainties in reporting, denominator and effective impact. Coverage clustering leads to zero dose children this while high numbers of doses are delivered there may still be pockets of low immunisation status which will influence the perceived benefits. Similarly, coverage estimates are based on population estimates that are themselves subject to substantial uncertainty. Finally, reporting mechanisms vary substantially between countries. It would be good to discuss and acknowledge these uncertainties.

-> Thank you for this very important feedback. We elaborated on this limitation and uncertainties associated with the data in our Discussion (Lines 397-404).

4. Over the years 2010-2019 most countries saw an improvement in achieved coverage- in line with this, the reduction seen for 2020 may appear more stark compared to the end of the time period rather than the average. Would it be feasible to compare a shorter initial time period to capture this variation ie comparing 2015-2019 to the pandemic year.

-> Thank you for this comment. Based on the visual inspection of the pre-trend between 2010-2019 (Figure 1), we did not observe a notable difference in the trend in immunization coverage between 2010-2015 and 2015-2019. To confirm this observation, we followed your suggestion and conducted the same analysis comparing the post-year (2020) to a shorter pre-period between 2015-2019. The result from these analyses is now available in the Supplementary Information (Tables S3-1 ~ S3-4). In summary, we observed similar results with very little difference in effect sizes for the difference-in-difference coefficient across all analyses conducted for different thresholds and covariate adjustments. We also referenced these results in the main manuscript to reinforce the robustness of our findings (Lines 258-260, 306-310).

5. Can you comment on the code availability. The link in the document does not appear to load.

-> Thank you for pointing this out. It is now made clear in the manuscript (Lines 260-263). We added the URL to the code and data repository as a text in the Supporting Information (S1, Paragraph 1), instead of hyperlink, with a list of references from where we acquired the data.

**Reviewer #3:** This study explores if the Covid 19 pandemic impact in 2020 on immunization coverage was less in 180 countries with stronger UHC.  It compares the coverage trends of 14 childhood vaccines between 2010 and 2020 between countries with higher UHC service coverage index score compared to low score (cut off 80 SCI). The comparison was compared using calendar year, income group, geographical region and preparedness index using global health security index (GHSI). 
The topic is relevant and timely. It adds evidence that stronger health systems are more resilient and that investments in UHC is important in advance of next pandemic
Suggest minor revisions for publication in PLOS Medicine. 

General questions are: 

1.    Why was the analysis controlled for by pandemic preparedness? In the IHR the index includes coverage. Could it be that preparedness could be well functioning in a country but health systems performing badly? I wonder if by controlling for it, the very thing you want to study also equals out because GHSI and SCI are linked. Table 3 shows no significance of any individual vaccine in the regression analysis. The explanation in the text instead says it is because of insufficient statistical power. Can you explain?

-> Thank you for this important question. We controlled for the pandemic preparedness using the GHSI 2019 to objectively account for country’s response capacity to the COVID-19 pandemic in the analysis. GHSI can be an important confounder to the analysis as it is correlated both to the UHC SCI (correlated through one of its 6 categories, not co-linear) and the change in immunization coverage during the pandemic. Regarding your question on the individual vaccine results, Table 3 is the summary of stratified DiD analyses for each vaccine type which reduced the sample size to at least 1/14^th^ vs. the main analysis for each analysis. The significantly reduced sample size supports our statement hypothesizing the non significant finding is due to insufficient power. To avoid confusion in interpretation, we added the explanation to the table legend in Table 3, pointing the readers to the Supporting Information where full analysis tables are available.

2.    The conclusion in the abstract to " strongly suggest policymakers to achieve UHC even during a public health crisis". Would suggest "achieve UHC in the coming years". The study did not study the possibility to achieve UHC during the pandemic.

-> Thank you for this suggestion. We modified the last sentence accordingly (Lines 447-449).

3.    Data is used from the end of 2020, but we know that the situation of impact on essential immunization was very dynamic during all of 2020, where Africa was extraordinary resilient compared to other Regions. Would it not have been good in include data on the epidemiologic situation or lockdown measures, which was very impactful reasons for impact on immunization services?

-> Thank you for this suggestion. We decided not to account for the varying impact of COVID-19 pandemic by country for two main reasons. First, we anticipated major challenge in coding the relevant covariates. For example, each country has varying surveillance and testing capacity, making the reported number of COVID-19 cases an inaccurate measure of the impact. Collecting exhaustive data on each country’s lockdown measures across all 180 countries similarly significantly compromises accuracy. Second, we thought the introduction of GHSI, to represent country’s epidemic/pandemic preparedness, could partially, but more objectively, account for countries’ response capacity to the COVID-19 pandemic.

4.    How was the “average coverage rate “ calculated? Was it an average of all 14 vaccines by country?

-> The average coverage rate is derived from the linear regression that includes all available data points from the countries for their administered vaccines.

Minor comments in addition:

1.    In table 1, why do you now mention the countries with less than 80 but more than 50 UHC SCI?

-> It is due to how we define the “treatment” and “control” group differently in the main analysis and the robustness check. In the main analysis, following the hypothesis “greater progress towards UHC, represented by the higher UHC SCI 2019 index, would safeguard countries’ ability to provide essential health services and ameliorate disruptions to service delivery in the face of public health crises like the COVID-19 pandemic”, treatment group is defined as “countries who achieved greater progress in UHC” (UHC SCI >= 80),” hence the control group is the countries with UHC SCI less than 80. Conversely, in the robustness check, we attempted to test the alternative hypothesis that “countries that made less progress towards UHC would be less able to manage disruptions to the delivery of essential health services during the pandemic,” hence the treatment group is defined as “countries who made less progress towards UHC (UHC SCI <50).” Therefore, the control group in this case is the countries with UHC SCI more than or equal to 50. Per your comment, we highlighted these differences in manuscript between Lines 188-200.

2.    HPV is not included in the Fig 1B. Was it used in the analysis? HPV coverage in 2020 was mainly affected by lockdowns and school closure which is less directly related to UHC.

-> Thank you for this comment. Immunization coverage data for HPV is collected separately from the rest of routine immunization coverage data for 14 childhood vaccines. We did not consider HPV in our analysis because: 1) HPV is mainly administered to adolescents through school and to adult populations; 2) is only administered to women in many countries; and 3) HPV data collection only covers ~100 countries from 2015 onwards.

3.    I find it a bit odd to describe in the discussion what equation to use in the next paper. Consider to avoid using the equation and explain plainly what is suggested.

-> Thank you for this suggestion. We excluded the equation from the Discussion and explained this approach in plain text (Lines 414-425)

From the academic editor: From the academic editor: The biggest concern is that the UHC index is highly correlated with national income (and health system spend)--so we may be actually assessing whether rich countries maintained immunization rather than whether UHC per se had an impact. To assess the latter, the authors could do an additional analysis of low-middle and low income countries only. Or split analysis into high income, middle income, and low-income. In any case, as written, I am not sure we can conclude much about UHC as the protective factor.

-> Thank you for this suggestion. We included the World Bank’s classification of country’s income group in all analyses to account for the potential confounding effect of country’s income (Methods described in Lines 157-158, 239-238). We also conducted additional sensitivity analysis by changing the UHC SCI cut-off values in sliding scale (Methods described in Lines 258-260 and 306-310, Results presented in Figure 3-1) that changes the composition of the countries in the treatment and control group to observe whether the confounding effect of income changes under different cut-off values. We observed the significant difference-in-difference coefficient across all analyses conducted using the cut-off value of 65 and above and concluded that while income is a significant confounder, the significant causal effect of high UHC persists, which is consistent with our initial findings.

Other editorial comments:

1. Title: Please revise your title according to PLOS Medicine's style.
-Your title must be nondeclarative and not a question.
-It should begin with main concept if possible.
-"Effect of" should be used only if causality can be inferred, i.e., for an RCT. Please place the study design ("A randomized controlled trial," "A retrospective study," "A modelling study," etc.) in the subtitle (ie, after a colon).
-Please use “sentence case capitalization” for the title. Please capitalize the first word of the subtitle. For example: “Universal healthcare coverage and childhood immunization coverage before and during the COVID-19 pandemic: A difference-in-difference analysis”

-> We revised our title following your style guide to make it nondeclarative. (Revised title: Universal Healthcare Coverage and Health Service Delivery in Times of Public Health Crises: A Difference-in-Difference Study of Childhood Immunization Coverage Before and During the COVID-19 Pandemic) (Lines 1-3)

2. Data availability statement: The Data Availability Statement (DAS) requires revision. Please update the data availability statement to indicate the where the full data sets and analysis code may be accessed.
For each data source used in your study:

a) If the data are freely or publicly available, note this and state the location of the data: within the paper, in Supporting Information files, or in a public repository (include the DOI or accession number).

b) If the data are owned by a third party but freely available upon request, please note this and state the owner of the data set and contact information for data requests (web or email address). Note that a study author cannot be the contact person for the data.

c) If the data are not freely available, please describe briefly the ethical, legal, or contractual restriction that prevents you from sharing it. Please also include an appropriate contact (web or email address) for inquiries (again, this cannot be a study author).

-> Thank you. We will update this when submitting the revised manuscript. It is also now stated in the Methods (Lines 260-263)

3. Throughout: Please avoid referring to “effect of universal healthcare coverage on childhood immunization coverage” and similar language that strongly implies causality. Please soften language to allow for the possibility of alternate explanations - identified associations may potentially provide support for a causal relationship.

-> Thank you for this feedback. We still believe it is important to articulate the main strength of the difference-in-difference design, which lies in its quasi-experimental nature that enables the observed findings to infer causality. (<https://www.annualreviews.org/doi/abs/10.1146/annurev-publhealth-040617-013507>) However, to avoid any strong implication or assertion and to allow for alternative explanations, we revised our language throughout the manuscript to soften the tone.

4. Abstract: Background: The final sentence should clearly state the study question.

5. Abstract: Methods and Findings: Please ensure that all numbers presented in the abstract are present and identical to numbers presented in the main manuscript text. Please include a brief mention of the inclusion criteria for the 180 countries, and please explicitly describe the main outcome measures. Please briefly describe how countries’ income group, geographical region, and pandemic preparedness were categorized/assessed. Please describe the threshold of UHC SCI >/=80) to establish the treatment and control groups, in terms of what this metric represents, and the rationale for selecting it.

6. Abstract: Methods and Findings: Please quantify the main results with 95% CIs and p values. Please refer to “statistically non-significant” findings. Please mention the important variables that are adjusted for in the analyses.

7. Abstract: Methods and Findings: In the last sentence of the Abstract Methods and Findings section, please describe the main limitation(s) of the study's methodology.

8. Abstract: Conclusions: * Please address the study implications without overreaching what can be concluded from the data; the phrase "In this study, we observed ..." may be useful. * Please interpret the study based on the results presented in the abstract, emphasizing what is new without overstating your conclusions. Please remove the “Funding” statement from the abstract.

-> We largely modified our abstract to accommodate all of the comments provided through #4-8.

9. Author summary: At this stage, we ask that you include a short, non-technical Author Summary of your research to make findings accessible to a wide audience that includes both scientists and non-scientists. The Author Summary should immediately follow the Abstract in your revised manuscript. This text is subject to editorial change and should be distinct from the scientific abstract. Please see our author guidelines for more information: <https://urldefense.proofpoint.com/v2/url?u=https-3A__journals.plos.org_plosmedicine_s_revising-2Dyour-2Dmanuscript-23loc-2Dauthor-2Dsummary&d=DwIGaQ&c=slrrB7dE8n7gBJbeO0g-IQ&r=XGzUN6S7CV-sOjf7f4TPQQ&m=4LEvEVNJJNON5B5y9Mq_5x7239xkMvIQzzH-zfddxTWgW8lGquJw-BFcuzVGhk4H&s=GIPA_6xjAYFRh1_rwFQtu2X0FCeTX7oxrbXpvMV1dhk&e=> 

-> We added the Author Summary as per the guideline immediately following the Abstract (Lines 47-73)

10. In-text references: Please use numbers within square brackets to refer to references within the main text. Please place brackets prior to sentence punctuation. Where multiple references are indicated, please do not include spaces within brackets.

-> The references and in-text citations are now compliant with the Vancouver style.

11. Main text: Please include line numbers running continuously throughout with the revised version.

-> We added line numbers to the revised manuscript and used them accordingly to point out the parts where revisions are made.

12. Introduction: Please provide more rationale and importance of the examination of childhood immunization rates specifically as representative of health service delivery, in general (for example, the public health implications of disruption in delivery of essential childhood vaccinations).

-> This rationale was originally provided in the beginning of the Methods section. We re-arranged the text so that it is now included in the Introduction (Lines 110-131)

13. Method: In the manuscript text, please indicate: (1) the specific hypotheses you intended to test, (2) the analytical methods by which you planned to test them, (3) the analyses you actually performed, and (4) when reported analyses differ from those that were planned, transparent explanations for differences that affect the reliability of the study's results. If a reported analysis was performed based on an interesting but unanticipated pattern in the data, please be clear that the analysis was data-driven.

-> Hypothesis is now articulated in Lines 134-137, analytical plan and the analyses performed is covered in Lines 168-253. The paragraph specifying the revisions during the peer review process is added between Lines 254-263.

14. Methods: Did your study have a prospective protocol or analysis plan? Please state this (either way) early in the Methods section.

a) If a prospective analysis plan (from your funding proposal, IRB or other ethics committee submission, study protocol, or other planning document written before analyzing the data) was used in designing the study, please include the relevant prospectively written document with your revised manuscript as a Supporting Information file to be published alongside your study, and cite it in the Methods section. A legend for this file should be included at the end of your manuscript.

b) If no such document exists, please make sure that the Methods section transparently describes when analyses were planned, and when/why any data-driven changes to analyses took place.

c) In either case, changes in the analysis-- including those made in response to peer review comments-- should be identified as such in the Methods section of the paper, with rationale.

-> We made clarifications by describing when the analysis was planned and conducted, and when the revision was made in the Methods section (Lines 254-263).

15. Methods: Please ensure that the study is reported according to the STROBE guideline, and include the completed STROBE checklist as Supporting Information. Please add the following statement, or similar, to the Methods: "This study is reported as per the Strengthening the Reporting of Observational Studies in Epidemiology (STROBE) guideline (S1 Checklist)."

The STROBE guideline can be found here: <https://urldefense.proofpoint.com/v2/url?u=http-3A__www.equator-2Dnetwork.org_reporting-2Dguidelines_strobe_&d=DwIGaQ&c=slrrB7dE8n7gBJbeO0g-IQ&r=XGzUN6S7CV-sOjf7f4TPQQ&m=4LEvEVNJJNON5B5y9Mq_5x7239xkMvIQzzH-zfddxTWgW8lGquJw-BFcuzVGhk4H&s=RzNUjfhhizxGMZ6j__b4BBx2Alh2Sj4LBGdDr3XV1dc&e=> 

When completing the checklist, please use section and paragraph numbers, rather than page numbers.

-> The STROBE checklist is now completed and added in the Supporting Information (S4). In the manuscript, we added a statement to indicate this addition in Lines 262-263.

16. Methods: Please provide additional detail for the UHC Service Coverage Index and 2019 Global Health Security Index (GHSI) in terms of what they represent and how they are incorporated into the analyses. Please also mention how geographical location was assigned/categorized.

-> “Data” subsection in the Methods (Lines 138-167) now describes both UHC SCI 2019 and GHSI 2019 in more detail. The text (Lines 244-245) now specifies how geographical location was categorized.

17. Methods: Page 6-7: “We hypothesized that greater progress towards UHC…” The use of “greater progress” in the text seems to imply changes in UHC SCI over time, rather than UHC SCI index at a single time point (2019). Please clarify.

-> This is now clarified in Lines 135-137.

18. Methods: Page 8: “For the DiD analysis, we leveraged the COVID-19 pandemic to introduce a “pre-post” variable wherein the years prior to 2020 were defined as “pre” (0) and the pandemic year of 2020 was defined as “post” (1).” Please specify the years.

-> This is now specified in Line 205.

19. Methods: Please specify the significance level used (eg, P<0.05, two-sided) and the statistical test used to derive a p value. When a p value is given, please specify the statistical test used to determine it.

-> All p-values are from the linear regression analyses.

20. Methods: Ethical approval: Please provide the name(s) of the institutional review board(s) that provided or waived ethical approval for the study.

-> Our study used the available data that does not include any private, identifiable information hence did not require IRB approval. We specified this in the Methods section (Lines 165-167)

21. Results: Please clarify “SD” in the text, at first use.

-> We clarified the SD at its first use in Line 275.

22. Results: Please quantify all results from the analyses with 95% CIs and p values. Where adjusted analyses are presented, please explicitly mention the covariates adjusted for, and please also provide the results of unadjusted analyses.

-> All tables now include 95% CIs and p-values. Unadjusted analyses are provided in the Supporting Informations and cited in the main text. Covariates in adjusted analyses are also explicitly mentioned in the titles and/or the legends.

23. Results: Page 12: “Using the DiD model, we found that countries a high UHC index (UHC SCI 2019 80) prevented a 2.93% reduction in overall childhood immunization coverage during the pandemic year of 2020 (p value= 0.01).” Please temper the use of language implying causality throughout, and please describe this association more clearly, avoiding interpretations such as “prevented” in the sentence. We suggest moving “This finding supports our hypothesis that greater progress towards UHC safeguard countries’ ability to provide essential health services, as measured by the proxy of childhood immunizations, during external shocks.” to the Discussion.

-> Thank you for this feedback. We still believe that it is important to articulate the main strength of the difference-in-difference study design, which lies in its quasi-experimental nature that enables the observed findings to infer causality. (<https://www.annualreviews.org/doi/abs/10.1146/annurev-publhealth-040617-013507>) However, to avoid any strong implication or assertion and to allow for alternative explanations, we revised our language throughout the manuscript to soften the tone.

24. Discussion: Please present and organize the Discussion as follows: a short, clear summary of the article's findings; what the study adds to existing research and where and why the results may differ from previous research; strengths and limitations of the study; implications and next steps for research, clinical practice, and/or public policy; one-paragraph conclusion.

-> Short summary is provided in Lines 364-379, followed by the added value of the study and how our study is the first of its kind (Lines 379-384). Strength and limitations are summarized in Lines 385-404, and next steps and suggestions for further improvement are elaborated in Lines 405-437, and a one-paragraph conclusion is provided in Lines 438-449.

25. References: Please use the "Vancouver" style for reference formatting, and see our website for other reference guidelines <https://urldefense.proofpoint.com/v2/url?u=https-3A__journals.plos.org_plosmedicine_s_submission-2Dguidelines-23loc-2Dreferences&d=DwIGaQ&c=slrrB7dE8n7gBJbeO0g-IQ&r=XGzUN6S7CV-sOjf7f4TPQQ&m=4LEvEVNJJNON5B5y9Mq_5x7239xkMvIQzzH-zfddxTWgW8lGquJw-BFcuzVGhk4H&s=yVyLG4Ozr66NjbOsrsCdM8JIcGOFyCrrRe1RwyAQ-YU&e=>

-> We updated our references and in-text citations to the “Vancouver” style with numbers in brackets.

26. Figures and Tables: Please ensure each figure and table (including supporting information tables) has a title and descriptive legend. Please define all abbreviations used in the figure/table within the legend.

-> We fixed all the titles and legends in the main text and the supplementary information to include abbreviations and make them more descriptive.

27. Table 2 and Table 3: Please include the covariates adjusted for in the legend, and please also present results from unadjusted analyses. Please present 95% CIs in addition to p values. When a p value is given, please specify the statistical test used to determine it.

-> We added 95% confidence intervals, included the base model, and referenced the Supplementary Information for unadjusted analyses. Legends are now fixed to include all covariates and abbreviations.

28. S1 Table: “Full dataset used for this analysis can be found here: LINK” Please provide the link to access the full data set, with a reference. Please update the data availability statement section of the manuscript submission system accordingly.

-> We added the URL as a text with a list of references from where we acquired the data. We also updated the data availability statement accordingly.

29. Table S2-1: Please specify the reference groups. Please mention the variables adjusted for in the analyses. Please also provide the 95% CIs, and results of unadjusted analyses.

-> We specified the reference groups and the variables adjusted for in the analyses and provided the 95% CIs, as well as the results of unadjusted analyses.

30. Table S2-2 through S2-14: Please mention the variables adjusted for in the analyses. Please also provide the 95% CIs, and results of unadjusted analyses. Please refer to p values as p<0.001 where applicable.

-> We specified the reference groups and the variables adjusted in the analyses. We provided the 95% CIs in all existing tables. P-value notations are now corrected as suggested. In addition, we provided additional tables tp present the results of the unadjusted analyses. As a result, the number of tables is now 27, with even-numbered tables for unadjusted analyses and odd-numbered tables for adjusted analyses.

31. Tables: We suggest including a table showing the characteristics of the countries included, summarizing UHC SCI (with thresholds of 80 and 50), income group, geographical location, GHSI.

-> We included two tables, namely Tables S1-3 and S1-4 (one for the cutoff value of 80 and another for 50, respectively), in the Supplementary Information and cited these tables in the main manuscript (Line 281)
