# Supplementary material for: Universal healthcare coverage and health service delivery before and during the COVID-19 pandemic: A difference-in-difference study of childhood immunization coverage from 195 countries
Source: PLoS Med. 2022 Aug 16;19(8):e1004060. doi: 10.1371/journal.pmed.1004060 (PMC9380914; doi:10.1371/journal.pmed.1004060)
Supplement: Attachment — Submitted filename: Response to editors.docx [file pmed.1004060.s007.docx]

**Response to Editors and Reviewers (Manuscript PMEDICINE-D-22-00227R1)**

Dear editors and reviewers,

On behalf of my co-authors, I would like to thank you for your constructive comments on our manuscript.

We followed your guidance and updated our manuscript to fulfill your suggestions and to meet PLOS Medicine’s requirements. We additionally confirm that this material has not and will not be submitted to any other journal for publication so long as this manuscript is under consideration at PLOS Medicine.

Please do not hesitate to reach out to me with questions.

Sincerely,

Yesim Tozan, PhD

1. Title: Please indicate the number of countries in the title.

-> It is now fixed (Line 3). The revised full title is “Universal Healthcare Coverage and Health Service Delivery in Times of Public Health Crises: A Difference-in-Difference Study of Childhood Immunization Coverage from 180 Countries Before and During the COVID-19 Pandemic”.

2. Short title: Please specify the “COVID-19 pandemic” or similar.

-> It is now fixed (Lines 4-5). The revised short title is “Universal healthcare coverage and health service delivery during the COVID-19 pandemic”.

3. Data availability statement: Thank you for including the github dataset. Please provide information on how to access the WHO/UNICEF Joint Estimates of National Immunization Coverage, The IHME UHC Service Coverage Index, World Bank Classification for income groups, and the GHSI for 2019. (The references provided in “S1 Dataset used for the analysis” might be useful).

-> Please see the updated data availability statement below:

Original data can be accessed through following sources.

- Global Health Security Index (GHSI) 2019: Hopkins U. Global Health Security (GHS) Index. Retrieved from: https://www ghsindex org/wp-content/uploads/2019/10/2019-Global-Health-Security-Index pdf. 2019.
- Universal Healthcare Coverage Service Coverage Index (UHC SCI) 2019: Institute for Health Metrics and Evaluation. Global Burden of Disease Study 2019 (GBD 2019) UHC Effective Coverage Index 1990-2019. In:2020.
- WHO/UNICEF Joint Estimates of National Immunization Coverage: UNICEF. Immunization. In:2021. Retrieved from: <https://data.unicef.org/topic/child-health/immunization/>
- Country income groups categorization: World Bank. The World by Income and Region. https://datatopics.worldbank.org/world-development-indicators/the-world-by-income-and-region.html. Published 2021. Accessed September 14, 2021, 2021.

All relevant data and analysis scripts uploaded in the github repository (https://github.com/sk9076/UHC_DID ). The path to the repository is available in Supporting Information files and is cited in the main manuscript.

4. Abstract: Line 24-25: We suggest “...quantified the relationship between UHC and childhood immunization coverage before and during the COVID-19 pandemic.”

-> It is not fixed (Lines 25-27).

5. Abstract: Please combine the Methods and Results sections into one section, “Methods and findings”.

-> Please find the merged section in Lines 25-41.

6. Abstract: Line 27: It may be helpful to mention that only 12 vaccines were considered in the final analyses.
-> It is not mentioned in Line 28 (“…provision 12 out of 14 childhood vaccines…”

7. Abstract: Line 35: Here, and throughout the manuscript, please replace “less reduction” with “smaller reduction” or similar.
-> We replaced “less reduction” and “less decline” across the manuscript to “smaller reduction” and “smaller decline”.

8. Abstract: Line 37: Rather than “control group” it would be helpful to explain these are the countries with UHC less than 80.

-> We updated the language to be “the countries with UHC index less than 80” (Lines 37-38).

9. Abstract: Lines 35-38: For both the analysis with UHC SCI > or = 80 and 50, please give the numbers of countries that fell above and below these thresholds.

-> The numbers of countries are now included in Line 36 and 39 respectively.

10. Abstract: Line 42: Please replace “less declines” with “smaller declines” or similar.
-> We replaced “less reduction” and “less decline” across the manuscript to “smaller reduction” and “smaller decline”.

11. Abstract: Line 43: Please reword this to avoid drawing conclusions about causality. “...may potentially provide support for the importance of UHC in building health system resilience.” or similar.

-> The sentence is now updated with your suggested wordings (Lines 45-45).

12. Author summary: Line 62-64: Please use “smaller decline” here. Please also describe the control group.
-> We replaced “less reduction” and “less decline” across the manuscript to “smaller reduction” and “smaller decline”.

We also replaced the “control group” with “the countries with UHC index less than 80” (Lines 63-66)

13. Author summary: Line 65-66: Please remove this point.
🡪 It is now removed.

14. Introduction: Line 101: Here and throughout, please remove spaces from within brackets [9,10].
-> We removed spaces from within the citation brackets throughout the manuscript.

15. Introduction: Line 128-131: Please revise throughout to remove emphasis on causal implications. “This goal of the study was to examine the relationship between UHC and health service delivery during the COVID-19 pandemic shock.” or similar may be useful.
-> The sentence is now updated to “The goal of this study was to examine the relationship between UHC and health service delivery during the COVID-19 pandemic shock.” (Lines 129-130)

16. Methods: Hypothesis: Please move the hypothesis to the end of the Introduction.
-> It is now moved to the end of the introduction (Lines 130-133).

17. Methods: Line 140: We suggest “These data include…” or “This dataset includes…” or similar.

-> It is now replaced with “This dataset includes..” (Line 139).

18. Methods: Line 143: Please remove spaces from within reference brackets.
-> We removed spaces from within the citation brackets throughout the manuscript.

19. Methods Line 164: The text indicates that compiled data is presented in paragraph 1 of S1 file. Please make it clear that this paragraph contains information to access the full dataset as well as the information about the original data.
-> The sentence is now updated to provide all suggested information. Please see the revised sentence below and in the manuscript (Lines 161-164):

“The Supporting Information presents the full list of countries included in the analysis (Table S1-1), as well as the compiled data from the aforementioned sources and the information on how to access the original data (Paragraph 1 in S1).”

20. Methods: Line 185-187: “Lastly, even in resource-limited countries with poor health service provisioning and lower UHC index values, childhood immunization programs are prioritized and perform relatively well compared to other service areas.” Please provide a reference for this sentence.

-> We cited the reference #1 and #14 to support this statement (Line 185).

21. Methods: Line 218-226: Please provide a brief explanation for why these particular vaccine/dose combinations were selected as indicators (e.g. why the third PCV dose, but the birth dose of Hep B?) if possible.

-> The 14 vaccine-dose combinations are considered the essential childhood immunizations and tracked systematically and longitudinally across 180 countries by the WHO-UNICEF Joint Estimate of Immunization Coverage and by the two agencies involved, WHO and UNICEF in this effort. Any further information or rationale was not provided by these agencies.

22. Methods: Data analysis: Please specify the significance level used (eg, P<0.05, two-sided) and the statistical tests used to derive a p value.
-> We added a sentence to state this in Lines 258-259:

“Throughout the analysis, all statistical tests are performed two-sided and we use the threshold of 0.05 (P<0.05) for stating the statistical significance.”

23. Results: Line 277: Here and throughout, it may be helpful to consistently refer to UHC index less than or greater/equal to 80 (similar to lines 273-276) rather than switch to “treatment” and “control” groups.

-> We updated this sentence to be “Of the 1,556 observations that took place during the pandemic year of 2020, countries with the high UHC index (UHC SCI 2019≥80) (N=204) had an average childhood immunization coverage rate of 91.9% (SD 9.4), and countries with the UHC index lower than 80 (N=1,352) had an average coverage rate of 81.7% (SD 16.1).” (Lines 277-279)

24. Results: Line 296-299: Although this is implied, it would be helpful to please also present here the Pre-post coefficient for UHC SCI greater than equal to 80 and less than 80, with 95% CIs and p values, to support that those countries with an index of 80 and greater experienced no significant change while the decline was significant in countries with an index of less than 80.
-> While Pre-post coefficient for countries with UHC index less than 80 can be directly interpreted from Table 2, along with its 95% CI and p value, Pre-post coefficient for countries with UHC SCI greater than equal to 80 is an interpretation of the joint effect of Pre-post variable and the Pre-post*Treatment variable. While we can present the joint effect size and the 95% CI by calculating the simple sum, calculation of p-value for this joint effect is not explicit based on the current analysis. We still provided the text explanation in Lines 298-300 but would recommend editors to consider not including this. We also provided an additional figure to visualize the differential drop in immunization coverage between two groups (Figure 2 in Line 308) based on the effect size and the 95% CI only.

25. Results: Line 303: Please use “not significant” here.

-> it is now fixed in Line 315)

26. Results: Line 306-310: “Subsequently, when we repeated the same analysis using the sliding threshold of UHC SCI between 50 and 80 with step increments of five, we observed a significant association between UHC and a differential drop in immunization coverage during the pandemic from the threshold value of 65 and above (Supporting Information Figure S3-1).” Please quantify these results in Figure S3-1, with the difference-in-difference coefficients, 95% CIs, and p values. Please indicate in the legend the tests used to determine significance.
-> To avoid the plot being crowded, we provided this information in the separate table (Table S3-1) and updated the reference to the Supporting Information accordingly in the manuscript (Lines 322-324).

27. Results: Line 340: Please use “absence of statistical significance” here.

-> It is now updated in Lines 352-353)

28. Discussion: Line 365-368: We suggest rewording to: “Using the DiD methodology to measure the differences in immunization coverage before and during the first year of the COVID-19 pandemic, we observed significantly less decline in childhood immunization coverage among countries with greater progress toward UHC.” or similar.

-> The sentence is now updated to be “Using the DiD methodology to measure the differences in immunization coverage before and during the first year of the COVID-19 pandemic, we observed significantly smaller decline in childhood immunization coverage among countries with greater progress toward UHC.” (Lines 378-381)

29. Discussion: Line 369: We suggest revising to: “Our findings suggest that countries with greater progress towards UHC were able to mitigate the decline in childhood immunization coverage…”
-> It is now updated (Lines 382-383)

30. Discussion: Line 375: Please revise to: “With our robustness checks, we observed that the association between UHC index and change in immunization coverage was only observed among countries with strong UHC performance…”
-> It is now updated (Lines 388-390)

31. Discussion: Line 379-381: Please revise to: “Nonetheless, to the best of our knowledge, our study is one of the first attempts to quantify the possible contribution of UHC in safeguarding health system performance during a public health crisis.”

-> It is now updated (Lines 393-395)

32. Discussion: Please be sure that the Discussion is organized as follows: a short, clear summary of the article's findings; what the study adds to existing research and where and why the results may differ from previous research; strengths and limitations of the study; implications and next steps for research, clinical practice, and/or public policy; one-paragraph conclusion
-> We double-checked that the discussion is organized in suggested order: a short, clear summary of the article's findings (Lines 377-383; what the study adds to existing research and where and why the results may differ from previous research (Lines 383-388); strengths and limitations of the study (Lines 389-418); implications and next steps for research, clinical practice, and/or public policy (Lines 419-451); one-paragraph conclusion (Lines 452-463).

33. References: Please use the "Vancouver" style for reference formatting, and see our website for other reference guidelines [https://urldefense.com/v3/__https://journals.plos.org/plosmedicine/s/submission-guidelines*loc-references__;Iw!!BhJSzQqDqA!Tzbe3m0To1qh2CviiCy88ScfrXclOJFhOBxMXqxpwk5jEtRlsuHNR2ZUgVB0oZpQIMDaeaxWRTejUg$](https://urldefense.com/v3/__https:/journals.plos.org/plosmedicine/s/submission-guidelines*loc-references__;Iw!!BhJSzQqDqA!Tzbe3m0To1qh2CviiCy88ScfrXclOJFhOBxMXqxpwk5jEtRlsuHNR2ZUgVB0oZpQIMDaeaxWRTejUg$) 

-Please pay particular attention to the journal title abbreviations (e.g. The Lancet should be Lancet in Reference 2 and throughout). -> this is now updated

-Please provide complete details for reference 12, reference 13, reference 15, reference 18. -> fixed

-Please change the journal title abbreviation to BMJ Glob Health for reference 17.-> fixed

-Please change the journal title abbreviation to Lancet Infect Dis for reference 27. -> fixed

-Please change the journal title abbreviation to Lancet Glob Health for reference 29. -> fixed

-Please change the journal title abbreviation to BMC Int Health Hum Rights for reference 34. -> fixed

-Please change the journal title abbreviation to PLOS Glob Public Health for reference 35. -> fixed

34. Table 1: It might be helpful to also include a section of the table listing those countries that fall between UHC SCI 80 and 50, as these would be the ones to “switch” from control to treatment groups between the analyses.

-> This is now included

35. Figure 1: Please define all abbreviations used in the legend.

-> This is now included

36. Supporting information Table S1-3: We suggest moving the table of summary characteristics for the countries to the main text of the manuscript.
-> We moved this to the manuscript and adjusted the table numbering and referencing accordingly (Lines 283-287)

37. Supporting information Tables S3-4: Please make it clear in the title and/or legend that for these analyses you are replicating using a pre period of 2015-2019.

-> It is not clarified in the legend

38. Supporting Information 4: STROBE Checklist: We suggest including this as a separate document.

-> It is now provided as a separate document
